# A Proteomics-Based Identification of the Biological Networks Mediating the Impact of Epigallocatechin-3-Gallate on Trophoblast Cell Migration and Invasion, with Potential Implications for Maternal and Fetal Health

**DOI:** 10.3390/proteomes11040031

**Published:** 2023-10-12

**Authors:** Yueh-Chung Chen, Chen-Chung Liao, Hao-Ai Shui, Pei-Hsuan Huang, Li-Jane Shih

**Affiliations:** 1 Department of Medicine, School of Medicine, National Defense Medical Center, Taipei 114201, Taiwan; chenyuehchung.tw@yahoo.com.tw; 2 Division of Cardiology, Department of Internal Medicine, Taipei City Hospital, Renai Branch, Taipei 106243, Taiwan; 3 Department of Health Promotion and Gerontological Care, Taipei University of Marine Technology, Taipei 111078, Taiwan; 4 Department of Special Education, University of Taipei, Taipei 100234, Taiwan; 5 Mass Spectrometry Facility, Instrumentation Resource Center, National Yang Ming Chiao Tung University, Taipei 112304, Taiwan; ccliao@nycu.edu.tw (C.-C.L.);; 6 Cancer Progression Research Center, National Yang Ming Chiao Tung University, Taipei 112304, Taiwan; 7 Graduate Institute of Medical Sciences, National Defense Medical Center, Taipei 114201, Taiwan; 8 Department of Medical Laboratory, Taoyuan Armed Forces General Hospital, Longtan, Taoyuan 325208, Taiwan

**Keywords:** EGCG, migration, invasion, proteomics, trophoblast cells

## Abstract

Trophoblast migration and invasion play crucial roles in placental development. However, the effects of (-)-epigallocatechin-3-gallate (EGCG) on trophoblast cell functions remain largely unexplored. In this study, we investigated the impact of EGCG on the survival of trophoblast cells and employed a proteomics analysis to evaluate its influence on trophoblast cell migration and invasion. Be-Wo trophoblast cells were treated with EGCG, and a zone closure assay was conducted to assess the cell migration and invasion. Subsequently, a proteomics analysis was performed on the treated and control groups, followed by a bioinformatics analysis to evaluate the affected biological pathways and protein networks. A quantitative real-time PCR and Western blot analysis were carried out to validate the proteomics findings. Our results showed that EGCG significantly suppressed the trophoblast migration and invasion at a concentration not affecting cell survival. The proteomics analysis revealed notable differences in the protein expression between the EGCG-treated and control groups. Specifically, EGCG downregulated the signaling pathways related to EIF2, mTOR, and estrogen response, as well as the processes associated with the cytoskeleton, extracellular matrix, and protein translation. Conversely, EGCG upregulated the pathways linked to lipid degradation and oxidative metabolism. The quantitative PCR showed that EGCG modulated protein expression by regulating gene transcription, and the Western blot analysis confirmed its impact on cytoskeleton and extracellular matrix reorganization. These findings suggest EGCG may inhibit trophoblast migration and invasion through multiple signaling pathways, highlighting the potential risks associated with consuming EGCG-containing products during pregnancy. Future research should investigate the impact of EGCG intake on maternal and fetal proteoforms.

## 1. Introduction

Green tea is a widely consumed beverage with a high concentration of epigallocatechin gallate (EGCG), the most abundant polyphenol catechin with the highest biological activity. In addition to being consumed in beverages, pure EGCG is often used as a self-care food supplement [1]. Numerous studies have demonstrated that EGCG has remarkable antioxidant, anti-inflammatory, and cell-protective properties, which can improve cell survival and protect cells against various harmful factors [2,3,4]. EGCG also has significant suppressive effects on certain cell activities, suggesting that it could potentially be used to treat disorders with abnormal cell behaviors. Indeed, several studies have shown that EGCG can suppress the proliferation, survival, and migration of various cancer cells [5,6,7], thus potentially serving as an adjuvant for treating cancer overgrowth and metastasis.

While the inhibition of cancer cell activities by EGCG is beneficial, the same effects could potentially affect the physiological functions of normal cells and cause negative outcomes. For pregnant women, consuming green tea or purified EGCG is not completely safe or risk-free, as EGCG has been shown to inhibit blastocyst implantation, cause embryonic cytotoxicity, suppress the neural progenitor cells in the embryo, and affect the development of the central nervous system of the fetus [8,9,10]. These side effects might arise from EGCG’s inhibitory effect on the cell activities required for fetal development, including cell division and migration, which are crucial for the morphogenesis and development of the fetus [8]. However, these studies were conducted in vivo and in vitro on murine models, and it is imperative that additional research is undertaken using human cells to provide a more comprehensive understanding of these potential effects. Trophoblast cells, the outer layer of cells in the blastocyst, play a crucial role in placental development to form an interface, i.e., the placenta, between the embryo and the uterine tissue of the mother. During the early stages of pregnancy, trophoblasts migrate and invade the sub-endometrial area and spiral arteries, disrupting the vascular structure of the spiral arteries and forming sinus-like structures [11,12]. Therefore, the factors that affect the migration and invasion of trophoblasts can impair the placental function and subsequent fetal development. The insufficient migration of trophoblast cells has been linked to preeclampsia, which is a severe disorder in pregnant women [13]. To date, the impacts of tea consumption remain a subject of debate within the scientific community. Certain studies have suggested that the intake of green tea may play a preventive role in the occurrence of preeclampsia, as indicated by research sources [14,15]. Conversely, opposing studies have presented findings that have linked tea consumption to an elevated risk of pregnancy-induced hypertension [16,17]. The question that persists is whether EGCG, the principal catechin found in green tea, exerts any influence on the processes of trophoblast migration and invasion.

So far, the effect of EGCG on the protein expression in trophoblast cells remains unclear. A proteomics analysis is a useful approach for unraveling the mechanisms underlying the various activities of EGCG [18]. It has been used to analyze the EGCG-regulated proteins in many types of cells, demonstrating that EGCG can regulate and target different sets of proteins that are dependent on different cell types and different experimental conditions [19,20,21]. However, to date, a proteomic analysis of EGCG-treated trophoblasts has never been performed, especially with regard to the effects of EGCG on trophoblast migration and invasion. In the absence of primary culture trophoblasts, Be-Wo cells are a well-established model of trophoblast cells for investigating cell invasion [22] and preeclampsia [23,24]. Therefore, the present study aimed to investigate whether EGCG can disturb the migration and invasion abilities of trophoblast cells, and, if so, to perform a proteomic analysis to investigate the expressional changes in the proteins underlying EGCG’s effects.

## 2. Materials and Methods

### 2.1. Chemical Reagents

The EGCG used was obtained from Sigma (St. Louis, MO, USA) and dissolved in 0.1% DMSO in the sterile medium for cell treatment. DMSO (0.1%) in a sterile medium without EGCG was used as a control. Penicillin-streptomycin, Ham’s F12K medium, FBS, and trypsin were purchased from GIBCO-BRL Life Technologies (New York, NY, USA).

### 2.2. Cell Culture

Be-Wo trophoblast cells (American Type Culture Collection, Rockville, MD, USA; ATCC- CCL-98TM) were maintained in Ham’s F12K medium supplemented with 15% heat-inactivated FBS, 0.5% penicillin, and 0.5% streptomycin. All the cells were maintained at a density of 5 × 10^5^ cells/cm^2^ in a humidified atmosphere of 95% air and 5% CO_2_ at 37 °C.

### 2.3. Zone Closure Assay for Cell Migration and Invasion

The Oris™ Cell Zone Closure Assay method (AMS Biotechnology, Abingdon, UK) was used to investigate the impact of EGCG on the migration and invasion of the Be-Wo trophoblast cells, according to the manufacturer’s protocol. Briefly, silicone stoppers were inserted into the test wells of the assay plate coated with collagen or basement membrane extract (BME) to make cell-free zones that excluded the cells from the central detection area of the wells. The Be-Wo trophoblast cells (10,000 cells/100 μL) were then seeded into the outer areas surrounding the silicone stoppers. Once a confluent monolayer was formed, the cells were treated with EGCG (10 μM) or left without treatment (0 μM) as controls. The silicone stoppers were then removed to expose the unseeded zone for the cells to move into the area. Assays were performed using either the Oris™ migration kit coated with collagen or the Oris™ invasion kit coated with a 3-D BME. The zone areas at the pre-migration (0 h) and after-migration (24 h) time points were analyzed using the Image analysis software MOSAIC (Version 1.6) (Tucsen Photonics, Fuzhou, Fujian, China). After treatment with EGCG for 24 h, the cell migration or invasion extents were estimated as a percentage of the zone closure using the following formula: “Zone closure percent = (Pre-closure area − after-closure area) × 100/Pre-closure area”.

### 2.4. Proteomics Comparison of EGCG-Treated and Control Trophoblast Cells

#### 2.4.1. Preparation and Digestion of Protein Samples

The Be-Wo trophoblast cells (1 × 10^6^), treated with or without EGCG (control), were suspended in PBS before being sonicated five times on ice. After sonication, the cell lysate was then spun at 14,000× *g* for 15 min at 4 °C to remove any cell debris. The resulting supernatants were then processed using a SMART Digest Kit (Thermo Fisher Scientific, Waltham, MA, USA) and cleaned of salt with ZipTip C18 microcolumns (Merck Millipore, Billerica, MA, USA). The resulting peptides were dried using a Speed-Vac and stored at −20 °C.

#### 2.4.2. Analysis of Protein Samples Using Liquid Chromatography-Mass Spectrometry

Each tryptic digest was resuspended in 20 μL of 0.1% formic acid and analyzed using a nanoLC-MS/MS system. The NanoAcquity system (Waters Corporation, Milford, MA, USA) was coupled to an Orbitrap Velos hybrid mass spectrometer with a nanoelectrospray ionization source (Thermo Fisher Scientific, Waltham, MA, USA). Peptide samples were separated using a BEH C18 column (25 cm 75 μm, Waters Corporation, Milford, MA, USA) with a segmented gradient of from 5% to 35% of solution B for 210 min, at a flow rate of 300 nL/min. The mobile phases were prepared as solution A (0.1% formic acid in water) and solution B (0.1% formic acid in acetonitrile). The eluted peptides were ionized with a spray voltage of 1.7 kV and introduced into the LTQ-Orbitrap Velos mass spectrometer. The mass spectrometer was operated in the positive ion mode using a data-dependent acquisition method (isolation width: 2.0 Da). The resolution of the full MS was set to 30,000 with an *m*/*z* range of 400. The mass spectrum data of the peptides were obtained using a full mass spectrometer survey scan (*m*/*z* range of 350–1600). According to the data-dependent acquisition method, the 10 most intense multiply charged ions (2+ and 3+) were selected for the MS/MS scan. Collision-induced dissociation (CID) was performed for the MS/MS scan.

#### 2.4.3. Identification and Quantification of Proteins

The MS raw data were analyzed using the Peaks7.5 Studio software for proteomics (Bioinformatics Solutions Inc., Waterloo, ON, Canada). The search was conducted against the UniProt human protein database which contained 192,901 protein sequences (http://www.uniprot.org/, accessed on 17 January 2020). The search parameters included a parent mass error tolerance of 50 ppm, fragment mass error tolerance of 0.8 Da, trypsin enzyme set, and allowed for oxidation on methionine (+15.99 Da) and the carbamidomethylation of cysteine (+57.02 Da) as variable modifications with two missed cleavages. The average local confidence was set at >80%, and a decoy database was used to calculate the false discovery rate, which was set to <1%. A protein was considered to be identified if at least one unique peptide was matched. Protein quantification using MS spectra counting was performed using in-house software Protein-Q (Version 6) [25,26]. The individual spectral counts of the peptides were first normalized using the total MS/MS spectrum count.

### 2.5. Bioinformatics Analysis and Data Visualization

Bioinformatics and data visualization tools were used to assess the functional biological pathways and protein networks in the trophoblasts impacted by the EGCG treatment, including an Ingenuity Pathway Analysis (IPA, www.qiagen.com/ingenuity, accessed on 6 February 2020), Gene Set Enrichment Analysis (GSEA, http://www.broadinstitute.org/gsea, accessed on 12 November 2020), and KEGG enrichment analyses using the ShinyGO web tool (http://bioinformatics.sdstate.edu/go/, accessed on 31 January 2023). The differentially expressed proteins between the EGCG-treated trophoblast cells and control cells were used as input seed proteins for the enrichment analysis and for generating the functional pathways and biological networks.

#### 2.5.1. IPA Network Analysis

For the IPA network analysis, the modulated proteins were grouped by their known relationships into the “Functional Analysis” and “Canonical Pathway Analysis” of a network, with the significance being calculated using Fisher’s exact test (*p*-value < 0.05) to determine the probability of each biological function. Hypothetical protein interaction clusters between the molecules were analyzed. IPA computed a *p*-score to rank the networks according to the differentially expressed proteins. The score was calculated as *p*-score = −log10 (*p*-value), which indicated the probability of matching the input proteins in a protein–protein interaction from the Ingenuity Knowledge Base by random chance.

#### 2.5.2. KEGG Enrichment Analysis Using the ShinyGO Web Tool

The KEGG enrichment analysis was performed using the ShinyGO web tool (http://bioinformatics.sdstate.edu/go/, accessed on 31 January 2023) [27]. The analysis was conducted on a list of the genes identified from our proteomics study. All the differentially expressed protein IDs were converted into Ensembl IDs, and then the Ensembl gene IDs were inputted into the ShinyGO program interface to access the KEGG databases for the retrieval of pathway diagrams. To perform the analysis, the query genes were mapped to all the gene IDs in the database. A statistical analysis was conducted to compare the genomic features of the query genes with the background genes in the genome, including the chromosomal distribution, and the underlying molecular pathways and functional categories associated with the query gene set were provided. The graphical visualization and enrichment results were retrieved online.

#### 2.5.3. GSEA Analysis

The Gene Set Enrichment Analysis was performed using the GSEA program (version 4.3.2) (http://www.broadinstitute.org/gsea, accessed on 12 November 2020) [28]. The MS data from the EGCG-treated cells were loaded into the GSEA software as an experimental dataset, while the MS data from non-related cells were loaded as a control dataset. The Hallmark gene sets with various pre-defined groups of genes that are associated with specific biological functions or pathways were obtained from the Molecular Signatures Database and used as references. The proteins were ranked based on their relative EGCG/control expression levels, and the final enrichment plots for the gene sets with significant differences were obtained.

### 2.6. Quantitative Real-Time PCR

RNA was extracted from the cultured Be-Wo cells using the Total RNA isolation kit (Cat. No. NA017-0100, GeneDireX, Inc., Taoyuan City, Taiwan), and cDNA was synthesized from 1 μg of total RNA using oligo-dT with the GeneDireX cDNA synthesis kit (Cat. No. MB305-0050, GeneDireX). The reaction mix was prepared with cDNA templates (2 ng), Fast SYBR^®^ Green Master Mix (2X, Cat. No. 4385612, Applied Biosystems, Thermo Fisher Scientific, Waltham, MA, USA), forward and reverse primers (each 1 μM), and RNase-free water. Then, 25 μL of the reaction mix was transferred into each well of a 96-well plate. The quantitative real-time PCR program was run with the experiment parameters on the Applied Biosystems StepOnePlus Real-Time PCR machine (Applied Biosystems, Thermo Fisher Scientific, Waltham, MA, USA). DataAssist Software (Version 2.3) (Applied Biosystems, Thermo Fisher Scientific, Waltham, MA, USA) was used for the data analysis, using the comparative CT (∆∆CT) method to calculate the relative quantitation of the gene expression. All the PCR primers are listed in Supplementary Table S1.

### 2.7. Western Blot Analysis

For the Western blot analysis, 30 μg of protein was separated using 12% sodium dodecyl sulfate (SDS) polyacrylamide gel electrophoresis with a gel-loading buffer. The separated proteins were then blotted onto polyvinylidene fluoride transfer membranes, followed by incubation with a specific primary antibody, including ABI2 (PA5-45199, Invitrogen), MMP2 (#4022, Cell Signaling, Danvers, MA, USA), or MMP9 (#3852, Cell Signaling). The immunoblots were then incubated with an appropriate secondary antibody (goat anti-rabbit IgG or donkey anti-goat IgG conjugated with horseradish peroxidase), and visualized by adding Western Lightning™ chemiluminescence reagent and captured with the Syngene PXi system. After normalization to the GAPDH protein levels, the levels of the target proteins were further normalized to the mean intensity of the corresponding bands in each group to compensate for potential variations in the protein loading and sample preparation.

### 2.8. Statistical Analysis

A Student’s t-test was performed to determine the significance of the differences in the protein expressions in the spectral count of the mass spectrometry, quantitative PCR, and Western blots. Protein data are expressed as mean RSD. Benjamini–Hochberg correction was performed to calculate the adjusted *p*-values of multiple comparisons of the normalized spectral count of the MS data, in order to reduce false discovery. For all the statistical tests, the level of significance was set at 0.05.

## 3. Results

### 3.1. EGCG Inhibits Migration and Invasion of Trophoblast Cells at a Non-Cytotoxic Concentration

**Figure 1:**
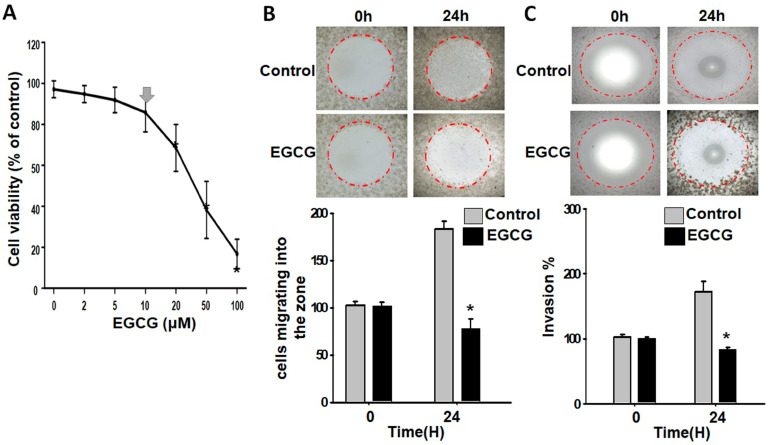
Effects of 10 μM EGCG on the survival, migration, and invasion of Be-Wo trophoblast cells. (**A**) Concentration-dependent cell survival curve detected using the MTT assay. Note that each concentration was subjected to six repetitions of the experiment, and that concentrations below 10 μM (the arrow on the plot) did not induce significant cytotoxic effects, but did cause dose-dependent reductions in cell viability. (**B**) Cell migration across collagen-coated wells was assessed using the Oris™ Cell Migration Assay kit. The migration of EGCG-treated (10 μM) Be-Wo cells was markedly decreased compared to that of control cells. (**C**) Cell invasion of a 3D extracellular matrix composed of a basement membrane extract was assessed using the Oris™ Cell Invasion Assay kit. Similar to migration, the invasion of EGCG-treated (10 μM) Be-Wo cells was also markedly decreased compared to that of control cells. The zone areas allowing the cells to either migrate or invade are marked with red circles. Note that the brighter areas within the red circles represent regions with a lower cell density, while the darkened areas within the red circles represent regions with a higher cell density. * *p* < 0.05 vs. the control group.

Previous studies have shown that a concentration of 20 μM of EGCG can inhibit the growth of trophoblast cells [29] To avoid interfering factors, we used Be-Wo cells as a model and lowered the EGCG concentration. The concentration-dependent survival curve in Figure 1A indicates that the EGCG did not have a significant cytotoxic effect on the Be-Wo trophoblast cells at 10 μM, despite the mild reduction in cell viability. We therefore chose this concentration for measuring the behaviors of living cells in the subsequent experiments.

Cell invasion and migration assays were conducted using the Oris zone closure method, which creates fixed areas of cell-free zones without damaging the surface protein matrix using silicone stoppers. As shown in Figure 1B, the EGCG-treated cells migrated at a slower rate compared to the non-treated cells after allowing them to migrate to the cell-free zone on a surface pre-coated with collagen proteins. Furthermore, as shown in Figure 1C, the EGCG-treated cells had a weaker ability to invade three-dimensional zones, which were coated with a 3D extracellular basement membrane, compared to the non-treated cells. These findings demonstrate that EGCG can considerably decrease the migration and invasion of trophoblast cells without compromising their viability.

### 3.2. Proteomics Analysis Reveals Significant Differences in Protein Expression between EGCG-Treated and Control Groups

**Figure 2:**
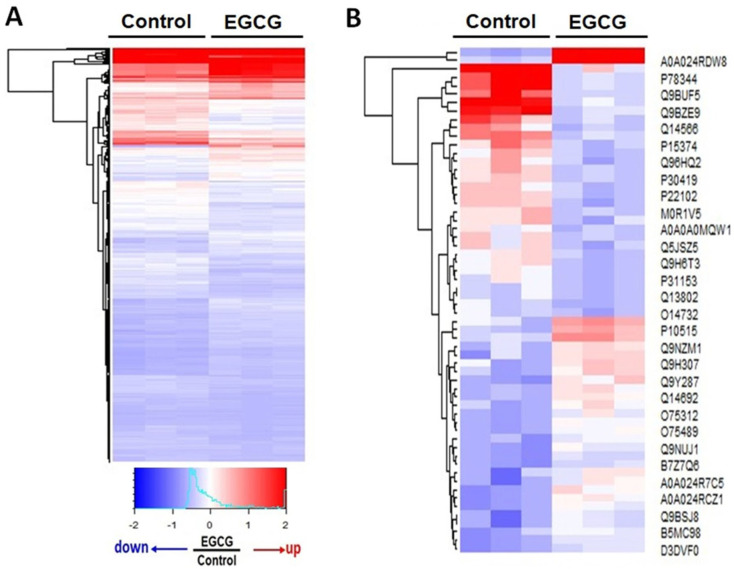
Heatmap representation of proteomics results comparing EGCG-treated and control groups. (**A**) The heatmap displays the differential expressional proteins (569 proteins) in the EGCG-treated and control groups, based on the z-score (−2–2) of normalized spectrum counts in MS. Each column represents a biological replicate, and each row represents a protein. The red color indicates upregulation in the EGCG-treated group, while the blue color indicates downregulation. The intensity of the color represents the magnitude of the z-score. Three biological replicates were used for each group. (**B**) Heatmap illustrating the top 30 upregulated and downregulated proteins in the EGCG-treated as compared to the control groups. Red coloration signifies a significant increase in protein expression, while blue denotes a decrease. The proteins names are displayed on the right side of the heatmap.

This study investigated the effects of EGCG on the protein expression related to altered migration and invasion behaviors. Two experimental conditions were evaluated: cells treated with and without EGCG, referred to as the “EGCG” and “control” groups. The EGCG group showed lower migration and invasion abilities compared to the control group. Proteomics analyses were conducted on both groups, and significant differences in protein expression were observed. The expressions of 569 proteins were found to be significantly altered by the EGCG treatment as compared to the control group (Supplementary Table S2). These findings suggest that EGCG may modify trophoblast migration and invasion by affecting the expressions of specific proteins (Figure 2).

### 3.3. Downregulation of EIF2, mTOR, and Estrogen Response Signaling Pathways and Protein Translation Processes in EGCG-Treated Trophoblast Cells

**Figure 3:**
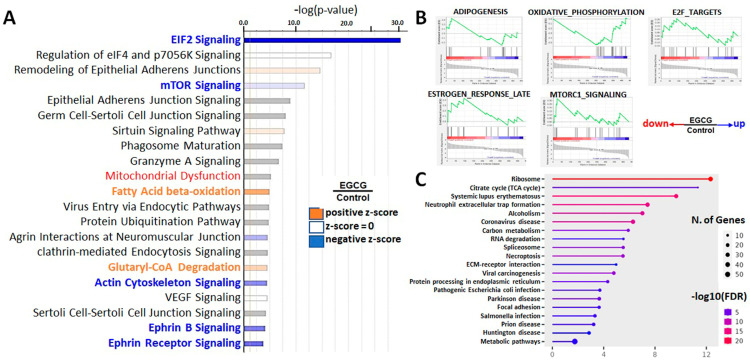
Enrichment analysis plots displaying sets of EGCG-regulated biological pathways in trophoblast cells. (**A**) IPA biological pathways plots with a *p*-score to indicate significantly EGCG-regulated proteins. Note that the EIF2 and mTOR signaling pathways were down-regulated (blue bars), whereas fatty acid beta-oxidation and glutaryl-CoA degradation pathways were upregulated (red bars). (**B**) GSEA curves of genes expressed in trophoblast cells. Each plot shows enrichment plots comparing gene expression in EGCG vs. controls. Note that the EIF2, mTOR, and estrogenresponse gene sets were downregulated, while the mitochondrial oxidative phosphorylation set was upregulated. (**C**) Enrichment of KEGG pathways generated by the ShinyGO web tool. The ribosome, TCA cycle, protein processes, ECM-receptor interaction, and focal adhesion were shown to be impacted by EGCG treatment.

An enrichment analysis helps to identify proteomes with specific disease phenotypes or biological functions from a large number of proteins. To further understand the effect of EGCG on the trophoblast cells, multiple enrichment analyses were performed, including an IPA, GSEA analysis, KEGG analysis (Figure 3) (Supplementary Figure S1).

The combined results of the IPA, GSEA, and GO enrichment analyses revealed that the activity of the EIF2 and mTOR signaling pathways were downregulated (EIF2 and mTOR sets in Figure 3A,B), affecting the protein translation. EIF2 signaling modulates protein synthesis in response to various stresses, while the mTOR signaling pathway coordinates multiple cellular processes and metabolism with environmental stresses [30,31]. As the protein expression of these two pathways was downregulated, EGCG-treated trophoblast cells might have a reduced ability to cope with external environmental changes. Moreover, the GSEA analysis showed that EGCG suppressed the response of cells to estrogen, as the proteins related to estrogen response were downregulated (Figure 3B).

### 3.4. Upregulation of Lipid Degradation, Oxidative Metabolism, and in EGCG-Treated Trophoblast Cells

EGCG upregulated the lipid aerobic metabolism (Fatty acid beta-oxidation set) and mitochondrial oxidative phosphorylation (OXIDATIVE_PHOSPHORYLATION set) (Figure 3A). These results are consistent with previous studies showing that EGCG increases fat metabolism and has beneficial effects on obesity and metabolic syndrome. However, excessive lipid metabolism may reduce cholesterol-related hormones and precursors for hormone production [32], which may affect the activity of trophoblast cells and their ability to respond to hormones during embryonic development and placental formation. This inference can be partially confirmed by the response of estrogen (HALLMARK_ESTROGEN_RESPONSE_LATE set, Figure 3A).

### 3.5. EGCG Modulated Protein Expression in Trophoblast Cells by Regulating Gene Transcription

**Figure 4:**
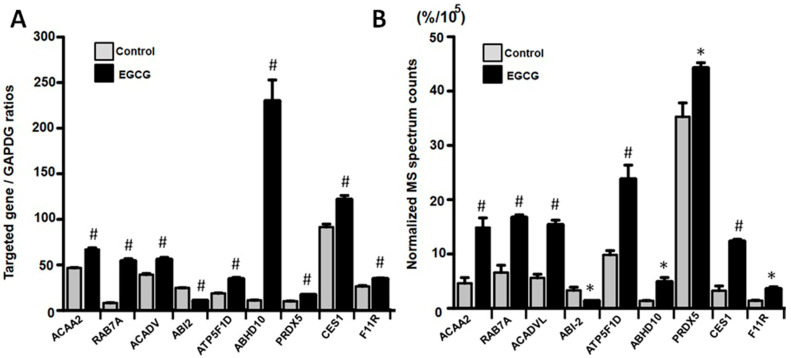
Validation of proteomics results using quantitative PCR. (**A**) Quantitative PCR verification of protein expression levels for randomly selected proteins (ACAA2, RAB7A, ACADVL, ABI2, ATP5F1D, ABHD10, PRDX5, CES1, and F11R) from various enrichment analysis categories. (**B**) Quantitative data from normalized spectrum counts were obtained through mass spectrometry. Note that trends of EGCG-induced gene expression changes measured using qPCR were consistent with those quantified using mass spectrometry, providing validation of the proteome analysis results and suggesting that differences in some protein expressions were due to differential gene regulation. * and # indicate *p* < 0.05 and *p* < 0.01 between EGCG and control groups. Note that untargeted MS-based proteomics provide relative, not absolute, quantification (normalized spectrum counts) of proteins along the axis.

Previous literature has demonstrated that EGCG can modulate protein expression levels through gene regulation. To gain a better understanding of the mechanism through which EGCG impacts protein expression, we selected representative hub proteins involved in key steps of the biological processes from each enrichment analysis category, including lipid metabolism (ACAA2 and ACADVL), intracellular transport (RAB7A), cytoskeleton dynamics (ABI2), energy metabolism (ATP5F1D), ABHD10 (post-translational modification), CES1 (xenobiotic and drug metabolism), and F11R (cell adhesion). We then confirmed whether the mRNA qPCR validation results were consistent with the changes observed in the mass spectrometry ion count quantitative data. Our qPCR results demonstrated that the differences in the expressions of these genes (Figure 4A), exhibiting a consistent trend of changes with the findings of the proteome analysis (Figure 4B), indicating that EGCG can broadly regulate the genes of trophoblast cells via transcription.

### 3.6. EGCG Suppresses Cytoskeletal and Extracellular Matrix Reorganization Proteins

**Figure 5:**
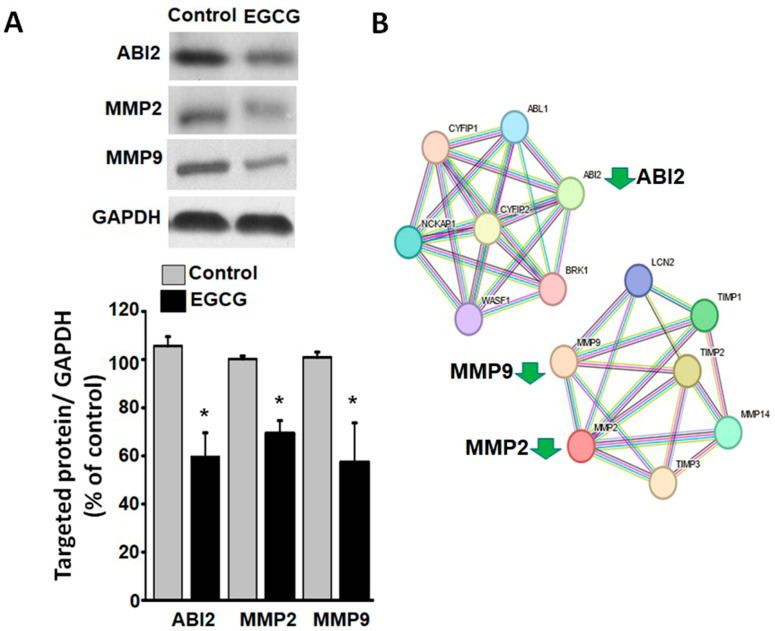
Impact of EGCG on protein expressions involved in cell migration and invasion demonstrated by Western blot analysis. (**A**) Western blot analysis confirms the EGCG-induced downregulation of proteins involved in the reorganization of the extracellular matrix and intracellular cytoskeleton in trophoblast cells. The upper panel displays representative blot images of ABI2, MMP2, MMP9, and GAPDH proteins in the lysates of cells. The lower panel displays statistical and quantification data normalized by GAPDH levels of corresponding proteins, followed by normalization to the mean intensity of the corresponding bands in each group. Data are presented as mean *±* standard deviation and * *p* < 0.05 vs. control group. (**B**) STRING analysis showing cytoskeleton-regulating networks with downregulation of ABI2 and extracellular matrix interaction networks with downregulation of MMP2 and MMP9.

Successful implantation and placental development rely on the ability of trophoblast cells to migrate and invade. However, our experiments demonstrated that the EGCG treatment significantly impaired the migration and invasion abilities of the trophoblast cells. To enable migration and invasion, cells must undergo a significant reorganization of their cytoskeletons. Our proteomic analysis revealed a downregulation of these reorganization processes, including the downregulation of ABI2 (ID: Q9NYB9), which plays a primary role in cytoskeleton reorganization [33]. Additionally, cells must undergo a significant reorganization of the extracellular matrix for migration and invasion. In terms of trophoblast cells, matrix metalloproteinase 2 and 9 (MMP2 and MMP9) are two key proteases capable of breaking down components of the extracellular matrix [34,35]. As confirmed by the Western blot analysis, EGCG downregulated ABI2, MMP2, and MMP9 in the trophoblast cells (Figure 5A). Furthermore, the bioinformatics analysis indicated that ABI2 and MMPs belong to two independent biological networks without direct interaction (Figure 5B).

## 4. Discussion

To date, EGCG has been shown to be beneficial for health as a result of its anti- inflammatory, antioxidative, and anticancer properties [1,2,3,4]. However, some studies have suggested that tea consumption may cause pregnancy anomalies and increase the risk of preeclampsia [16,17]. The present study demonstrated that EGCG can significantly inhibit the migration and invasion of trophoblast cells at a concentration that does not adversely affect cell survival.

In order for the placenta to develop, trophoblast cells must migrate and invade, which requires extensive cytoskeletal remodeling and the degradation of the extracellular matrix. For trophoblast cells to change their shape, their actin dynamics must change, as well as their secretion of proteases, such as MMP2 and MMP9, for the degradation of extracellular matrix components [33,34,35]. This study found that EGCG inhibited the expression of ABI2, a protein that plays a critical role in actin dynamics. ABI2 is localized to the actin polymerization sites in protrusive membrane structures and interacts with other actin-associated proteins to modulate the cell morphology [36,37]. A further finding of our study was that EGCG can decrease the expressions of MMP2 and MMP9. The matrix metalloproteinases MMP2 and MMP9 are enzymes that facilitate cell migration and invasion by degrading the extracellular matrix [34,35]. Therefore, the EGCG-mediated downregulation of MMP2 and MMP9 may impair extracellular matrix degradation and compromise trophoblast cell migration and invasion.

Furthermore, our proteomics analysis revealed that EGCG affects multiple signaling pathways and biological pathways in trophoblast cells. The EIF2 signaling pathway is crucial to regulating the global and specific mRNA translation in response to a variety of environmental stresses. In conjunction with activated mTOR signaling, EIF2α phosphorylation is triggered in response to various stresses and amino acid starvation, thereby supporting essential cellular functions such as growth, differentiation, survival, and cell mobility [30,31]. These two pathways are highly interdependent, especially for controlling metabolism under stressful conditions [38]. The results from our study indicated that EGCG downregulated both HALLMARK E2F TARGETS, as well as HALLMARK MTORC1 SIGNALING. The E2F and mTORC1 pathways play important roles in regulating the ribosome biosynthesis, as well as the protein synthesis, in trophoblast cells during placental development, as well as the migration and invasion of cells [39,40]. A reduction in the expressions of these two signaling pathways may have a significant impact on normal trophoblast function. Due to its impact on these signaling pathways, EGCG appears to impair the stress response and adaptive capacity of trophoblast cells when exposed to environmental stressors during placentation and development [39,40,41].

Another finding of our study was that EGCG enhanced the mitochondrial oxidative phosphorylation, as well as the fatty acid beta-oxidation, in trophoblast cells. This is consistent with previous studies showing that EGCG increases the oxidative metabolism in adipocytes, which is beneficial for obesity and metabolic syndrome, as it reduces the burden caused by excessive fat [42,43]. However, an excessive oxidative metabolism may have a detrimental effect on trophoblast cells, as it was noticed that EGCG also had an impact on the late estrogen response pathway in trophoblast cells, since acetyl CoA is a precursor for both mitochondrial oxidative phosphorylation and cholesterol synthesis. An excessive oxidative metabolism can negatively impact the production of steroid hormones by overusing the precursor for cholesterol synthesis [32]. It is known that trophoblast cells secrete protein and steroid hormones to stabilize the placental activity during implantation. There is a need for further research to determine whether EGCG disrupts the steroid metabolism of trophoblast cells.

Recent advances in proteomics research have led to the introduction of the term “proteoform” to characterize the various forms and variants of proteins resulting from genetic polymorphisms, RNA splice variants, and post-translational modifications [44,45]. One of the limitations of our study is that, while we investigated the protein-level effects of EGCG on trophoblast cell migration and invasion, we did not investigate the specific proteoforms implicated. Another limitation of our study is that, although Be-Wo cells have been accepted as a cell model of trophoblasts for studying invasion and preeclampsia [22,23,24], the cells have a carcinogenic origin, and some effects may be a consequence of carcinogenesis. Future research could concentrate on elucidating the proteoform-level alterations in primary trophoblast cells induced by EGCG treatment, as this could shed light on the molecular mechanisms underlying its effects on trophoblast cells.

## 5. Conclusions

**Figure 6:**
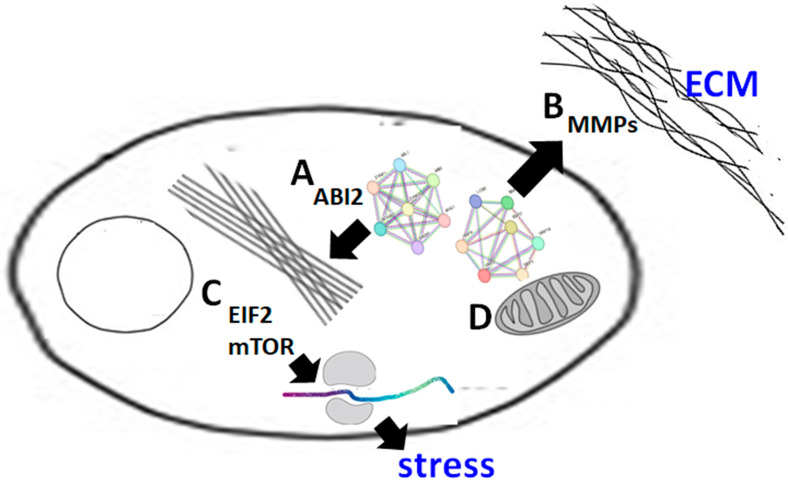
Schematic diagrams of biological pathways and networks underlying the effects of EGCG in trophoblast cells. Using the bioinformatics analysis tools, the impact of ECGC on trophoblast cells can be summarized into four different functional clusters: (A) downregulation of cytoskeletal reorganization, (B) downregulation of ECM reorganization, (C) downregulation of ribosome-associated signaling for environmental stresses, and (D) upregulation of oxidative metabolism in mitochondria.

In summary (Figure 6), our study provides insight into the potential mechanisms through which EGCG influences trophoblast cell migration and invasion through the regulation of the critical proteins involved in cytoskeletal remodeling and extracellular matrix degradation. In addition, EGCG may impair trophoblast function by downregulating the ribosome-associated signaling and modifying the oxidative metabolism in mitochondria. The potential association of EGCG with the development of placenta and preeclampsia needs to be clarified by further research.

## Data Availability

All the data and materials in the current study are available upon reasonable request.

## Supplementary Materials

The following supporting information can be downloaded at: https://www.mdpi.com/article/10.3390/proteomes11040031/s1, Figure S1: The top four signaling networks from the IPA analysis; Table S1: List of primers used in Quantitative Real-Time PCR; Table S2: List of proteomics-identified proteins differentially expressed between EGCG-treated and control trophoblast cells.
