# Supplementary material for: A Proteomics-Based Identification of the Biological Networks Mediating the Impact of Epigallocatechin-3-Gallate on Trophoblast Cell Migration and Invasion, with Potential Implications for Maternal and Fetal Health
Source: Proteomes. 2023 Oct 12;11(4):31. doi: 10.3390/proteomes11040031 (PMC10594419; doi:10.3390/proteomes11040031)
Supplement: Supplementary file 1 [file proteomes-11-00031-s001.zip › Supplementary Table S2.pdf]

Table S2: List of proteomics-identified proteins differentially expressed between EGCG-treated and control trophoblast cells. A total of 569 proteins were altered after EGCG treatment, with their accession number, name, gene ID, mass (Da), fold change, and adjusted p-value listed in the table. Benjamini-Hochberg correction was performed to calculate the adjusted p-values of multiple comparisons of normalized MS data.

Protein Accession number	Protein Name	Gene Name	Expected Mass (Daltons)	Fold Change	Adjusted p-Value
A4D198	Similar to mKIAA0038 protein	LOC392647	32604	0.431569	0.0009
P25705	ATP synthase subunit alpha, mitochondrial	ATP5F1A	59751	1.754337	0.0013
Q0VAS5	Histone H4	HIST1H4H	11314	1.918819	0.0013
Q8N532	TUBA1C protein	TUBA1C	36649	0.56201	0.0013
E7ETC0	Nucleolysin TIAR	TIAL1	14978	0.421041	0.0013
O60814	Histone H2B type 1-K	H2BC12	13890	1.725299	0.0013
P57053	Histone H2B type F-S	H2BC12L	13944	1.725299	0.0013
P58876	Histone H2B type 1-D	H2BC5	13936	1.725299	0.0013
P62807	Histone H2B type 1-C/E/F/G/I	H2BC10	13906	1.725299	0.0013
Q0D2M2	HIST1H2BC protein	HIST1H2BC	13834	1.725299	0.0013
Q5QNW6	Histone H2B type 2-F	H2BC18	13920	1.725299	0.0013
Q93079	Histone H2B type 1-H	H2BC9	13892	1.725299	0.0013
Q99877	Histone H2B type 1-N	H2BC15	13922	1.725299	0.0013
Q99879	Histone H2B type 1-M	H2BC14	13989	1.725299	0.0013
Q99880	Histone H2B type 1-L	H2BC13	13952	1.747294	0.0016
P06899	Histone H2B type 1-J	H2BC11	13904	1.776083	0.0020
P23527	Histone H2B type 1-O	H2BC17	13906	1.776083	0.0020
P33778	Histone H2B type 1-B	H2BC3	13950	1.776083	0.0020
Q16778	Histone H2B type 2-E	H2BC21	13920	1.776083	0.0020
M0R210	40S ribosomal protein S16	RPS16	14419	0.639981	0.0020
Q9BRP8	Partner of Y14 and mago	PYM1	22656	0.714169	0.0023
P10909	Clusterin	CLU	52495	2.63936	0.0023

P06576	ATP synthase subunit beta, mitochondrial	ATP5F1B	56560	1.53925	0.0023
V9HW31	ATP synthase subunit beta	HEL-S-271	56560	1.53925	0.0023
P33991	DNA replication licensing factor MCM4	MCM4	96558	0.344667	0.0023
P32119	Peroxiredoxin-2	PRDX2	21892	0.670185	0.0023
V9HW12	Epididymis secretory sperm binding protein Li 2a	HEL-S-2a	21892	0.670185	0.0023
P68363	Tubulin alpha-1B chain	TUBA1B	50152	0.462059	0.0023
F8WBG8	Drebrin-like protein	DBNL	13665	0.785918	0.0023
Q9UHD8	Septin-9	SEPTIN9	65401	1.18557	0.0024
E9PBF6	Lamin-B1	LMNB1	44643	1.338649	0.0025
P09874	Poly [ADP-ribose] polymerase 1	PARP1	113084	1.571572	0.0025
P52272	Heterogeneous nuclear ribonucleoprotein M	HNRNPM	77516	1.203397	0.0027
Q9BSD0	GLUD2 protein	GLUD2	29290	2.484859	0.0031
O75616	GTPase Era, mitochondrial	ERAL1	48350	0.28181	0.0032
Q8N257	Histone H2B type 3-B	H2BC26	13908	1.806998	0.0033
Q1KSF8	XTP3TPA-transactivated protein 1	XTP3TPATP1	23626	0.321823	0.0033
G3XAL0	Malate dehydrogenase	MDH2	24595	1.206227	0.0033
A0A024R972	Laminin, gamma 1 (Formerly LAMB2), isoform CRA_a	LAMC1	174011	1.440805	0.0033
P07910	Heterogeneous nuclear ribonucleoproteins C1/C2	HNRNPC	33670	1.675478	0.0033
O95721	Synaptosomal-associated protein 29	SNAP29	28970	0.622089	0.0033
Q92882	Osteoclast-stimulating factor 1	OSTF1	23787	0.622089	0.0033
E7ENZ3	T-complex protein 1 subunit epsilon	CCT5	53848	0.672139	0.0033
A0A024R2S1	Microtubule-associated protein 4, isoform CRA_b	MAP4	63452	0.555358	0.0035
Q14376	UDP-glucose 4-epimerase	GALE	38282	0.135323	0.0035
Q9P2N5	RNA-binding protein 27	RBM27	118718	0.465468	0.0035
A0A087WYS3	Protein kinase C-binding protein 1	ZMYND8	121253	4.041536	0.0035
O00566	U3 small nucleolar ribonucleoprotein	MPHOSPH10	78864	1.876109	0.0035

	protein MPP10				
Q13868	Exosome complex component RRP4	EXOSC2	32789	1.690857	0.0035
V9HW37	Epididymis secretory protein Li 69	HEL-S-69	59671	0.707	0.0036
A0A075B746	28S ribosomal protein S21, mitochondrial	MRPS21	10689	1.3997	0.0036
Q9UPY8	Microtubule-associated protein RP/EB family member 3	MAPRE3	31982	1.3997	0.0036
P0C0S5	Histone H2A.Z	H2AZ1	13553	2.151065	0.0037
Q71UI9	Histone H2A.V	H2AZ2	13509	2.151065	0.0037
F6U211	40S ribosomal protein S10	RPS10	19868	0.61677	0.0037
Q6IS14	Eukaryotic translation initiation factor 5A-1-like	EIF5AL1	16773	0.442479	0.0038
A0A024R8V0	Septin 9, isoform CRA_a	SEPT9	63633	1.260864	0.0039
Q53T99	Ribosome biogenesis protein WDR12	WDR12	47708	1.753892	0.0039
O95433	Activator of 90 kDa heat shock protein ATPase homolog 1	AHSA1	38274	0.401424	0.0039
J3QRL5	Growth factor receptor-bound protein 2	GRB2	17565	0.326496	0.0039
Q5T624	Nuclear autoantigenic sperm protein	NASP	45793	0.500581	0.0039
P23141	Liver carboxylesterase 1	CES1	62521	3.789824	0.0039
P16401	Histone H1.5	H1-5	22580	2.192059	0.0040
P17858	ATP-dependent 6-phosphofructokinase, liver type	PFKL	85018	0.311915	0.0041
Q14692	Ribosome biogenesis protein BMS1 homolog	BMS1	145807	3.793924	0.0042
P30419	Glycylpeptide N-tetradecanoyltransferase 1	NMT1	56806	0.286968	0.0042
A0A024RCZ1	LEM domain containing 2, isoform CRA_a	LEMD2	56975	6.55978	0.0042
Q05639	Elongation factor 1-alpha 2	EEF1A2	50470	0.64409	0.0043
P22102	Trifunctional purine biosynthetic protein adenosine-3	GART	107767	0.267197	0.0044
Q9BUF5	Tubulin beta-6 chain	TUBB6	49857	0.235234	0.0044

Q01105	Protein SET	SET	33489	0.501989	0.0044
A8K5K5	Eukaryotic translation initiation factor 3 subunit G	EIF3G	35601	0.516606	0.0044
J7M2B1	Tyrosine-protein kinase receptor	EZR-ROS1	98949	0.700036	0.0044
A0A024R394	Cysteine and histidine-rich domain (CHORD)-containing 1, isoform CRA_c	CHORDC1	37534	0.279949	0.0044
B3KPA6	Acyl-Coenzyme A dehydrogenase, very long chain, isoform CRA_e	ACADVL	62582	2.733359	0.0044
P30084	Enoyl-CoA hydratase, mitochondrial	ECHS1	31387	1.617351	0.0044
B8ZZY2	Arf-GAP domain and FG repeat-containing protein 1	AGFG1	56413	0.645559	0.0044
J3KT51	Hematological and neurological-expressed 1 protein	JPT1	11012	0.701735	0.0044
Q9BZE9	Tether containing UBX domain for GLUT4	ASPSCR1	60183	0.225146	0.0044
H0YKK0	Small nuclear ribonucleoprotein polypeptide A', isoform CRA_a	SNRPA1	17673	1.304187	0.0044
B7ZW00	COL6A3 protein	COL6A3	278209	1.977024	0.0044
P49454	Centromere protein F	CENPF	367764	1.783265	0.0044
A0A0F7RPW6	D-dopachrome tautomerase 2	DDT2	14195	0.69985	0.0044
A6NHG4	D-dopachrome decarboxylase-like protein	DDTL	14195	0.69985	0.0044
J3KQ18	D-dopachrome decarboxylase	DDT	14193	0.69985	0.0044
J3QSB5	60S ribosomal protein L36	RPL36	10789	0.69985	0.0044
P56385	ATP synthase subunit e, mitochondrial	ATP5ME	7933	0.69985	0.0044
A0A024RBU0	Vacuolar protein sorting 37B (Yeast), isoform CRA_b	VPS37B	31307	0.482295	0.0044
O75489	NADH dehydrogenase [ubiquinone] iron-sulfur protein 3, mitochondrial	NDUFS3	30242	4.052613	0.0044
P02462	Collagen alpha-1(IV) chain	COL4A1	160615	40.84269	0.0044
Q9H6T3	RNA polymerase II-associated protein 3	RPAP3	75719	0.254783	0.0045
Q14696	LDLR chaperone MESD	MESD	26077	1.581738	0.0046

Q7L1Q6	Basic leucine zipper and W2 domain-containing protein 1	BZW1	48043	0.503508	0.0048
P68366	Tubulin alpha-4A chain	TUBA4A	49924	0.358497	0.0048
Q9Y520	Protein PRRC2C	PRRC2C	316911	0.309557	0.0049
P83105	Serine protease HTRA4	HTRA4	50979	3.036489	0.0050
P62917	60S ribosomal protein L8	RPL8	28025	0.678233	0.0050
A8MUH2	ATP synthase-coupling factor 6, mitochondrial	ATP5PF	13954	1.790254	0.0050
Q96J85	C-Mpl binding protein	LARP4	42505	0.361212	0.0050
A0A024R904	Calcyclin binding protein, isoform CRA_a	CACYBP	26210	0.595729	0.0050
H9ZYJ1	Negative elongation factor E	NELF-E	43242	0.415995	0.0050
C9JGE3	EWS RNA-binding protein variant 6	EWSR1	65135	0.400105	0.0050
A0A024RAJ9	Arylsulfatase B, isoform CRA_a	ARSB	59687	2.018292	0.0050
Q2TU89	Aging-associated protein 1	AAG1	79614	0.217306	0.0050
A0A1E1ERW3	Diaphanous homolog 1	DIAPH1	133881	0.309198	0.0052
V9HVZ7	Epididymis luminal protein 176	HEL-176	25036	1.302933	0.0052
P40855	Peroxisomal biogenesis factor 19	PEX19	32807	0.667177	0.0053
P04908	Histone H2A type 1-B/E	H2AC4	14135	1.844136	0.0053
Q7L7L0	Histone H2A type 3	H2AC25	14121	1.844136	0.0053
Q93077	Histone H2A type 1-C	H2AC6	14105	1.844136	0.0053
Q9NQ39	Putative 40S ribosomal protein S10-like	RPS10P5	20120	0.535232	0.0053
Q9NPJ3	Acyl-coenzyme A thioesterase 13	ACOT13	14960	3.078495	0.0055
A0A024R3X4	Heat shock 60kDa protein 1 (Chaperonin), isoform CRA_a	HSPD1	61055	1.813639	0.0055
P07954	Fumarate hydratase, mitochondrial	FH	54637	1.225252	0.0055
A3R0T8	Histone 1, H1e	HIST1H1E	21865	1.423702	0.0055
P10412	Histone H1.4	H1-4	21865	1.423702	0.0055
P16402	Histone H1.3	H1-3	22350	1.423702	0.0055
P63241	Eukaryotic translation initiation factor 5A-1	EIF5A	16832	0.540483	0.0055

A0A158RFU6	RAB7, member RAS oncogene family, isoform CRA_a	RAB7A	23490	2.528086	0.0055
A0A024R4Z0	Catenin (Cadherin-associated protein), delta 1, isoform CRA_c	CTNND1	68031	0.649775	0.0057
P14780	Matrix metalloproteinase-9	MMP9	78458	0.54116	0.0057
I3L3M7	Thioredoxin domain-containing protein 17	TXNDC17	9076	0.371766	0.0057
A0A087WWU9	HCG2039564, isoform CRA_a	SRP19	11850	0.51103	0.0057
Q9H8Y8	Golgi reassembly-stacking protein 2	GORASP2	47145	1.604051	0.0058
Q8NI61	Ribosomal protein S2	OK/KNS-cl.7	21721	0.535811	0.0058
Q13885	Tubulin beta-2A chain	TUBB2A	49907	0.550182	0.0058
Q9NZM1	Myoferlin	MYOF	234709	3.450851	0.0058
B4DKV7	Epidermal growth factor receptor kinase substrate 8-like protein 1	EPS8L1	73315	0.603522	0.0058
P23396	40S ribosomal protein S3	RPS3	26688	0.680663	0.0058
A0A024RDV0	HCG1811699, isoform CRA_a	hCG_1811699	87188	2.532097	0.0058
Q08E77	UTP14, U3 small nucleolar ribonucleoprotein, homolog C (Yeast)	UTP14C	87187	2.532097	0.0058
A0A024QZ30	Succinate dehydrogenase [ubiquinone] flavoprotein subunit, mitochondrial	SDHA	72692	3.082271	0.0058
C9IZU8	Ribulose-phosphate 3-epimerase	RPE	24916	0.387823	0.0058
P22061	Protein-L-isoaspartate(D-aspartate) O-methyltransferase	PCMT1	24636	0.649247	0.0058
P61024	Cyclin-dependent kinases regulatory subunit 1	CKS1B	9660	0.466859	0.0058
H3BLZ8	Probable ATP-dependent RNA helicase DDX17	DDX17	80440	0.788274	0.0058
O15514	DNA-directed RNA polymerase II subunit RPB4	POLR2D	16311	1.861207	0.0058
Q13011	Delta(3,5)-Delta(2,4)-dienoyl-CoA isomerase, mitochondrial	ECH1	35816	1.839644	0.0058
A0A024R370	TATA element modulatory factor 1, isoform CRA_a	TMF1	122842	1.878543	0.0058

A0A024RDW8	Collagen, type IV, alpha 2, isoform CRA_a	COL4A2	167553	18.25963	0.0058
Q0VD83	Apolipoprotein B receptor	APOBR	114874	1.865029	0.0058
Q9NS13	Placenta apolipoprotein B48 receptor type 2	APOB48R	113432	1.865029	0.0058
P78344	Eukaryotic translation initiation factor 4 gamma 2	EIF4G2	102362	0.204557	0.0058
Q9UQE7	Structural maintenance of chromosomes protein 3	SMC3	141542	3.115539	0.0058
R4GMU7	60S ribosomal protein L7-like 1	RPL7L1	22746	1.763266	0.0059
Q96L21	60S ribosomal protein L10-like	RPL10L	24519	0.585252	0.0059
Q52LJ0	Protein FAM98B	FAM98B	37191	0.684873	0.0059
P15586	N-acetylglucosamine-6-sulfatase	GNS	62082	1.542081	0.0059
D6RAT0	40S ribosomal protein S3a	RPS3A	25887	0.631128	0.0059
H3BM89	60S ribosomal protein L4	RPL4	37657	0.795086	0.0059
P09234	U1 small nuclear ribonucleoprotein C	SNRPC	17394	0.445263	0.0060
B8ZZ99	Coiled-coil domain-containing protein 115	CCDC115	19056	4.225769	0.0060
Q6NZ55	60S ribosomal protein L13	RPL13	24265	0.529964	0.0060
P31689	DnaJ homolog subfamily A member 1	DNAJA1	44868	0.481582	0.0060
K9JA46	Epididymis luminal secretory protein 52	EL52	84660	0.485734	0.0061
P07900	Heat shock protein HSP 90-alpha	HSP90AA1	84660	0.485734	0.0061
Q15181	Inorganic pyrophosphatase	PPA1	32660	0.709651	0.0061
V9HWB5	Epididymis secretory sperm binding protein Li 66p	HEL-S-66p	32660	0.709651	0.0061
H3BNC6	Cadherin-1	CDH1	59400	4.421494	0.0061
B4E321	Protein OS-9	OS9	46302	2.005803	0.0061
P17987	T-complex protein 1 subunit alpha	TCP1	60344	0.53834	0.0062
E7EN95	Filamin-B	FLNB	256281	0.764958	0.0062
A0A1B0GUA1	Alpha-aminoadipic semialdehyde dehydrogenase	ALDH7A1	56466	1.451513	0.0062

A0A024R228	Heterogeneous nuclear ribonucleoprotein K, isoform CRA_d	HNRPK	51219	0.725253	0.0062
P61978	Heterogeneous nuclear ribonucleoprotein K	HNRNPK	50976	0.725253	0.0062
F8W7F7	Transmembrane emp24 domain-containing protein 4	TMED4	20118	2.540429	0.0062
M0R0M7	Syntaxin-binding protein 2	STXBP2	45873	0.310874	0.0062
E5RJX2	40S ribosomal protein S20	RPS20	6828	0.642499	0.0062
Q5BJH1	PSAP protein	PSAP	26309	0.352291	0.0062
B2R491	40S ribosomal protein S4	RPS4X	29598	0.638961	0.0062
P04179	Superoxide dismutase [Mn], mitochondrial	SOD2	24722	1.244178	0.0062
O00391	Sulfhydryl oxidase 1	QSOX1	82578	3.896266	0.0062
P0C0S8	Histone H2A type 1	H2AC11	14091	1.66348	0.0062
P20671	Histone H2A type 1-D	H2AC7	14107	1.66348	0.0062
Q16777	Histone H2A type 2-C	H2AC20	13988	1.66348	0.0062
Q6FI13	Histone H2A type 2-A	H2AC18	14095	1.66348	0.0062
Q96KK5	Histone H2A type 1-H	H2AC12	13906	1.66348	0.0062
Q99878	Histone H2A type 1-J	H2AC14	13936	1.66348	0.0062
Q9BTM1	Histone H2A.J	H2AJ	14019	1.66348	0.0062
Q15393	Splicing factor 3B subunit 3	SF3B3	135577	1.954401	0.0064
Q5RKV6	Exosome complex component MTR3	EXOSC6	28235	1.409048	0.0065
Q15417	Calponin-3	CNN3	36414	0.643039	0.0065
K7EKQ5	Galectin-3-binding protein	LGALS3BP	22699	2.265051	0.0065
Q9H307	Pinin	PNN	81614	3.715002	0.0065
B7Z4L4	Dolichyl-diphosphooligosaccharide--protein glycosyltransferase subunit 1	RPN1	49921	2.465027	0.0066
O95833	Chloride intracellular channel protein 3	CLIC3	26648	0.500774	0.0066
P30041	Peroxiredoxin-6	PRDX6	25035	0.755668	0.0066
V9HWC7	Epididymis secretory sperm binding protein Li 128m	HEL-S-128m	25035	0.755668	0.0066

K7ESG5	Proteasome activator complex subunit 3	PSME3	23259	0.267647	0.0066
Q69YH5	Cell division cycle-associated protein 2	CDCA2	112676	1.618848	0.0066
M0R1H5	40S ribosomal protein S11	RPS11	9486	0.446351	0.0067
P16403	Histone H1.2	H1-2	21365	1.400582	0.0067
O00469	Procollagen-lysine,2-oxoglutarate 5-dioxygenase 2	PLOD2	84686	1.470393	0.0067
Q9H0L4	Cleavage stimulation factor subunit 2 tau variant	CSTF2T	64437	1.839654	0.0067
A0A024R0Q4	Phospholipase D family, member 3, isoform CRA_b	PLD3	54705	3.7817	0.0067
Q96HE7	ERO1-like protein alpha	ERO1A	54393	1.341454	0.0067
M0QZN2	40S ribosomal protein S5	RPS5	14763	0.683979	0.0067
Q15075	Early endosome antigen 1	EEA1	162466	0.787943	0.0068
E9PPT0	40S ribosomal protein S2	RPS2	21023	0.58807	0.0069
Q9NUW4	BRIX	BXDC2	15296	1.880213	0.0069
A0A024R5K1	Coronin	CORO1B	54235	1.200293	0.0069
K7EQK2	Hypoxia up-regulated protein 1	HYOU1	75356	1.357309	0.0069
A0A024RAV5	V-Ki-ras2 Kirsten rat sarcoma viral oncogene homolog, isoform CRA_b	KRAS	21425	0.624178	0.0069
F5H5G4	WW domain-binding protein 11	WBP11	23163	3.476259	0.0071
A0A0K0K1H7	Aconitate hydratase, mitochondrial	HEL-S-284	85565	1.335921	0.0072
Q5GGW2	40S ribosomal protein S18	RPS18	3908	0.711651	0.0072
M0R1V5	Epidermal growth factor receptor substrate 15-like 1	EPS15L1	11935	0.284329	0.0072
Q27J81	Inverted formin-2	INF2	135624	0.339626	0.0073
Q32MZ4	Leucine-rich repeat flightless-interacting protein 1	LRRFIP1	89253	0.586787	0.0075
Q15427	Splicing factor 3B subunit 4	SF3B4	44386	0.7035	0.0075
A0A0C4DGV4	Hepatitis B virus x interacting protein	LAMTOR5	18159	2.708541	0.0076
Q96GQ7	Probable ATP-dependent RNA helicase DDX27	DDX27	89835	2.43794	0.0077

P30042	ES1 protein homolog, mitochondrial	GATD3	28170	2.400626	0.0077
Q13724	Mannosyl-oligosaccharide glucosidase	MOGS	91918	0.414045	0.0077
Q58F09	Glucosidase I	GCS1	91907	0.414045	0.0077
Q92541	RNA polymerase-associated protein RTF1 homolog	RTF1	80313	0.414045	0.0077
A4D0Y7	Similar to 40S ribosomal protein S2	LOC392781	21069	0.567495	0.0078
P29350	Tyrosine-protein phosphatase non-receptor type 6	PTPN6	67561	0.507129	0.0078
A0A0B4J2C3	Translationally-controlled tumor protein	TPT1	22574	0.654982	0.0078
F8VP73	Biogenesis of lysosome-related organelles complex 1 subunit 1	BLOC1S1	15996	0.333182	0.0078
P04080	Cystatin-B	CSTB	11140	0.799829	0.0078
A0A024R141	Cathepsin L2, isoform CRA_a	CTSL2	37329	2.569225	0.0078
O60911	Cathepsin L2	CTSV	37329	2.569225	0.0078
D3DWK1	Eukaryotic translation elongation factor 1 delta	EEF1D	71422	0.679948	0.0078
V6A6X0	MHC class I antigen	HLA-C	41027	2.09267	0.0078
Q8TCS8	Polyribonucleotide nucleotidyltransferase 1, mitochondrial	PNPT1	85951	2.374842	0.0078
Q5CAQ4	TNF receptor-associated protein 1	TRAP1	57234	1.556048	0.0078
Q8WUW1	Protein BRICK1	BRK1	8745	0.428905	0.0079
Q00403	Transcription initiation factor IIB	GTF2B	34833	2.134958	0.0079
E9KL48	Epididymis tissue sperm binding protein Li 18mP	GLUD1	61398	1.907574	0.0079
P63220	40S ribosomal protein S21	RPS21	9111	0.792472	0.0080
A0A0J9YXN7	Perilipin-4	PLIN4	136020	0.462605	0.0080
P53999	Activated RNA polymerase II transcriptional coactivator p15	SUB1	14395	0.416125	0.0080
Q6IBA2	PC4 protein	PC4	14395	0.416125	0.0080
Q68D58	Putative uncharacterized protein DKFZp686M2226	DKFZp686M2226	32971	0.600413	0.0080
P35580	Myosin-10	MYH10	228999	0.846574	0.0080

D6PAV9	Intersectin 1 short form A variant 2	ITSN1	116617	0.427297	0.0080
A0A024R0L6	Platelet-activating factor acetylhydrolase, isoform Ib, gamma subunit 29kDa, isoform CRA_a	PAFAH1B3	25734	0.378475	0.0081
Q99988	Growth/differentiation factor 15	GDF15	34140	2.682671	0.0081
Q5T9B7	Adenylate kinase isoenzyme 1	AK1	23411	0.511691	0.0081
P15531	Nucleoside diphosphate kinase A	NME1	17149	0.642504	0.0081
A0A0A0MSK5	Torsin-1A-interacting protein 1	TOR1AIP1	52406	1.82291	0.0081
A0A024RBR1	Restin	RSN	160990	0.606127	0.0081
P30622	CAP-Gly domain-containing linker protein 1	CLIP1	162246	0.606127	0.0081
A0A024RD82	Methylmalonyl CoA mutase isoform 1	MUT	83134	1.882785	0.0081
P22033	Methylmalonyl-CoA mutase, mitochondrial	MMUT	83134	1.882785	0.0081
Q9BUH6	Protein PAXX	PAXX	21640	0.7171	0.0081
A0A024R7W5	YTH domain family, member 3, isoform CRA_a	YTHDF3	58311	1.706227	0.0081
Q8N3V9	Putative uncharacterized protein DKFZp451A052	DKFZp451A052	58190	1.706227	0.0081
P55268	Laminin subunit beta-2	LAMB2	195981	1.658336	0.0082
Q9GZR7	ATP-dependent RNA helicase DDX24	DDX24	96332	4.606492	0.0082
O15230	Laminin subunit alpha-5	LAMA5	399737	2.723596	0.0083
B5BTZ8	Small nuclear ribonucleoprotein polypeptide B''	SNRPB2	25458	2.197265	0.0083
P12268	Inosine-5'-monophosphate dehydrogenase 2	IMPDH2	55805	0.420881	0.0083
Q9Y2W1	Thyroid hormone receptor-associated protein 3	THRAP3	108666	1.61771	0.0083
O95295	SNARE-associated protein Snapin	SNAPIN	14874	0.499865	0.0083
P30049	ATP synthase subunit delta, mitochondrial	ATP5F1D	17490	2.411073	0.0084
E5KRK5	Mitochondrial NADH-ubiquinone	NDUFS1	79468	5.617638	0.0084

	oxidoreductase 75 kDa subunit				
P20042	Eukaryotic translation initiation factor 2 subunit 2	EIF2S2	38388	0.224965	0.0084
Q68DU0	Putative uncharacterized protein DKFZp781O2021	DKFZp781O2021	104819	0.663745	0.0084
P37802	Transgelin-2	TAGLN2	22391	0.656578	0.0084
P38117	Electron transfer flavoprotein subunit beta	ETFB	27844	1.807017	0.0084
P51970	NADH dehydrogenase [ubiquinone] 1 alpha subcomplex subunit 8	NDUFA8	20105	2.027524	0.0084
P61513	60S ribosomal protein L37a	RPL37A	10275	0.595445	0.0085
B3KUB9	SWAP-70 protein, isoform CRA_b	SWAP70	62034	0.593946	0.0085
J3QL01	60S ribosomal protein L38	RPL38	7977	0.575251	0.0085
Q9Y5X3	Sorting nexin-5	SNX5	46816	0.62919	0.0085
P17844	Probable ATP-dependent RNA helicase DDX5	DDX5	69148	0.687177	0.0085
Q8WX93	Palladin	PALLD	150564	0.20457	0.0085
A0MZ66	Shootin-1	SHTN1	71640	0.518385	0.0086
Q9Y3I0	tRNA-splicing ligase RtcB homolog	RTCB	55210	0.463833	0.0086
P40939	Trifunctional enzyme subunit alpha, mitochondrial	HADHA	83000	1.408096	0.0086
A0A024R9D2	Metadherin, isoform CRA_a	MTDH	63837	1.480306	0.0086
K7EME0	3-ketoacyl-CoA thiolase, mitochondrial	ACAA2	36283	3.225517	0.0086
P23528	Cofilin-1	CFL1	18502	0.688755	0.0086
V9HWI5	Cofilin 1 (Non-muscle), isoform CRA_b	HEL-S-15	18502	0.688755	0.0086
P08253	72 kDa type IV collagenase	MMP2	73882	0.681234	0.0086
P27635	60S ribosomal protein L10	RPL10	24604	0.619107	0.0086
Q4VXU2	Polyadenylate-binding protein 1-like	PABPC1L	68392	0.539912	0.0087
A0A024R0R4	SUMO-1 activating enzyme subunit 1, isoform CRA_b	SAE1	38450	0.531762	0.0088
Q3KNV1	KRT7 protein	KRT7	15033	1.724955	0.0088

O60568	Procollagen-lysine,2-oxoglutarate 5-dioxygenase 3	PLOD3	84785	7.516871	0.0088
K7EMA7	60S ribosomal protein L23a	RPL23A	7923	0.684465	0.0089
G3XAM7	Catenin (Cadherin-associated protein), alpha 1, 102kDa, isoform CRA_a	CTNNA1	92722	0.786502	0.0089
V9HW26	ATP synthase subunit alpha	HEL-S-123m	59751	1.42643	0.0090
Q6DHZ8	Activity-dependent neuroprotector homeobox	ADNP	123447	2.832805	0.0091
A1L3A7	Nuclear fragile X mental retardation protein interacting protein 2	NUFIP2	76121	0.657423	0.0091
P18583	Protein SON	SON	263830	1.847787	0.0092
V9HW84	Epididymis secretory sperm binding protein Li 124m	HEL-S-124m	73780	1.431026	0.0092
Q9HCN8	Stromal cell-derived factor 2-like protein 1	SDF2L1	23598	1.354432	0.0092
G3V0E4	Mitochondrial-processing peptidase subunit beta	PMPCB	54192	0.578803	0.0092
P15374	Ubiquitin carboxyl-terminal hydrolase isozyme L3	UCHL3	26183	0.2956	0.0092
P32969	60S ribosomal protein L9	RPL9	21863	0.606694	0.0095
A0A0K0K1L8	Epididymis secretory sperm binding protein Li 129m	HEL-S-129m	28723	0.802638	0.0095
Q06323	Proteasome activator complex subunit 1	PSME1	28723	0.802638	0.0095
P38432	Coilin	COIL	62608	0.289035	0.0095
P24666	Low molecular weight phosphotyrosine protein phosphatase	ACP1	18042	0.655843	0.0095
A0A024R7C5	Mitochondrial ribosomal protein L4, isoform CRA_a	MRPL4	34919	5.876567	0.0096
A0A024R261	HCG24487, isoform CRA_c	hCG_24487	21397	0.583121	0.0096
A0A0A6YYL6	Protein RPL17-C18orf32	RPL17-C18orf32	26373	0.583121	0.0096
P18621	60S ribosomal protein L17	RPL17	21397	0.583121	0.0096
O43598	2'-deoxynucleoside 5'-phosphate N-hydrolase 1	DNPH1	19108	0.350824	0.0097

E5RH77	40S ribosomal protein S14	RPS14	14295	0.593908	0.0098
A0A0C4DGQ5	Calpain small subunit 1	CAPNS1	33786	0.390453	0.0098
F8W1I5	Myosin light chain 6B	MYL6B	19036	0.414294	0.0098
C9JMV9	ABHD14A-ACY1 readthrough	ABHD14A-ACY1	56436	0.534138	0.0099
Q03154	Aminoacylase-1	ACY1	45885	0.534138	0.0099
V9HWA0	Aminoacylase	HEL-S-5	45885	0.534138	0.0099
Q9H0D6	5'-3' exoribonuclease 2	XRN2	108582	0.488121	0.0100
A0MNN4	CDW3/SMU1	SMU1	57544	2.078163	0.0100
G3V153	Caprin-1	CAPRIN1	70353	0.648715	0.0101
A0A075B7A0	60S ribosomal protein L18	RPL18	16198	0.588761	0.0101
A0A087X1B7	Chromatin target of PRMT1 protein	CHTOP	8520	1.640611	0.0101
P62277	40S ribosomal protein S13	RPS13	17222	0.577674	0.0101
P13995	Bifunctional methylenetetrahydrofolate dehydrogenase/cyclohydrolase, mitochondrial	MTHFD2	37895	2.124424	0.0101
P00966	Argininosuccinate synthase	ASS1	46530	0.471795	0.0101
Q5T6L4	Argininosuccinate synthase 1 isoform 1	ASS	46530	0.471795	0.0101
C9JDE9	3-ketoacyl-CoA thiolase, peroxisomal	ACAA1	40269	0.572587	0.0103
Q96HS1	Serine/threonine-protein phosphatase PGAM5, mitochondrial	PGAM5	32004	2.525425	0.0103
E9PDQ8	Succinate--CoA ligase [GDP-forming] subunit beta, mitochondrial	SUCLG2	41438	2.034429	0.0104
A0A024R8H9	HCG1985901	hCG_1985901	88893	3.194911	0.0109
P38646	Stress-70 protein, mitochondrial	HSPA9	73681	1.419001	0.0111
A0A024R4H5	DNAation factor, 45kDa, alpha polypeptide, isoform CRA_b	DFFA	29411	0.519088	0.0111
P82909	28S ribosomal protein S36, mitochondrial	MRPS36	11466	1.660895	0.0111
P30837	Aldehyde dehydrogenase X, mitochondrial	ALDH1B1	57206	2.26835	0.0111
Q9H2R7	NPD011	NPD011	24353	0.251759	0.0111

P42126	Enoyl-CoA delta isomerase 1, mitochondrial	ECI1	32816	2.302531	0.0112
E7EPA6	Laminin subunit beta-1	LAMB1	95142	0.773622	0.0112
Q9UQC2	GRB2-associated-binding protein 2	GAB2	74458	0.470477	0.0112
A0A024R2W4	Dystroglycan 1 (Dystrophin-associated glycoprotein 1), isoform CRA_a	DAG1	97541	1.835146	0.0112
P40222	Alpha-taxilin	TXLNA	61891	0.501711	0.0112
E9PKG6	Nucleobindin-2	NUCB2	40369	1.59082	0.0112
A0A024RB02	PTPRF interacting protein, binding protein 1 (Liprin beta 1), isoform CRA_a	PPFIBP1	96965	0.434035	0.0112
A0A024RAW0	Mediator complex subunit 21 isoform 1	SURB7	15564	2.563273	0.0112
F5H872	Mediator of RNA polymerase II transcription subunit 21	MED21	17278	2.563273	0.0112
K7ESJ4	Programmed cell death protein 5	PDCD5	7468	0.347047	0.0112
Q9NVJ2	ADP-ribosylation factor-like protein 8B	ARL8B	21539	0.347047	0.0112
G5E972	Lamina-associated polypeptide 2, isoforms beta/gamma	TMPO	46307	1.334238	0.0112
Q15149	Plectin	PLEC	531791	1.649113	0.0113
H3BND4	Pyridoxal-dependent decarboxylase domain-containing protein 1	PDXDC1	88762	0.381374	0.0113
B7Z6T4	RD RNA binding protein, isoform CRA_a	RDBP	38398	0.359786	0.0113
D3DVA8	Ubiquilin 4, isoform CRA_a	UBQLN4	46026	1.31188	0.0115
A0A024R7E3	DNA (cytosine-5)-methyltransferase	DNMT1	189567	0.520739	0.0115
A5D8V6	Vacuolar protein sorting-associated protein 37C	VPS37C	38659	0.561896	0.0116
P26599	Polypyrimidine tract-binding protein 1	PTBP1	57221	1.201714	0.0117
O75312	Zinc finger protein ZPR1	ZPR1	50925	3.407502	0.0117
A8MUD9	60S ribosomal protein L7	RPL7	24433	0.662136	0.0118
P14174	Macrophage migration inhibitory factor	MIF	12476	0.643082	0.0118
A0A024RBG7	Nuclear transcription factor Y, beta, isoform CRA_c	NFYB	22831	0.79394	0.0118

P61011	Signal recognition particle 54 kDa protein	SRP54	55705	0.564367	0.0118
A0A0U1RQK7	Eukaryotic translation initiation factor 4 gamma 3	EIF4G3	195272	0.623837	0.0118
Q9Y287	Integral membrane protein 2B	ITM2B	30338	8.679404	0.0118
Q02818	Nucleobindin-1	NUCB1	53879	1.345968	0.0118
Q9UJZ1	Stomatin-like protein 2, mitochondrial	STOML2	38534	1.884891	0.0118
A0A024R5S5	Eukaryotic translation initiation factor 3 subunit J	EIF3S1	29062	0.494874	0.0118
D6RIV2	OCIA domain-containing protein 1	OCIAD1	15392	2.170318	0.0119
Q96DA6	Mitochondrial import inner membrane translocase subunit TIM14	DNAJC19	12499	0.505931	0.0120
A0A1B0GUS4	HCG1789360	UBE2L5	17875	0.621264	0.0121
V9HW05	Epididymis luminal protein 210	HEL-210	10856	0.747928	0.0122
A0A024QYX3	RNA binding motif (RNP1, RRM) protein 3, isoform CRA_c	RBM3	17170	0.736004	0.0122
A0A140VJL3	Testicular tissue protein Li 89	HPRT1	24579	0.359237	0.0122
Q86VP6	Cullin-associated NEDD8-dissociated protein 1	CAND1	136376	0.39461	0.0122
A0A024R755	Calumenin, isoform CRA_a	CALU	37135	1.54205	0.0122
A0A024R6C4	Numb homolog (Drosophila), isoform CRA_a	NUMB	69431	0.685696	0.0122
P60763	Ras-related C3 botulinum toxin substrate 3	RAC3	21379	0.419159	0.0123
P31153	S-adenosylmethionine synthase isoform type-2	MAT2A	43661	0.20616	0.0123
Q9NUJ1	Mycophenolic acid acyl-glucuronide esterase, mitochondrial	ABHD10	33933	5.267589	0.0123
Q99523	Sortilin	SORT1	92068	2.130734	0.0124
O60271	C-Jun-amino-terminal kinase-interacting protein 4	SPAG9	146205	0.750003	0.0124
Q8WYP5	Protein ELYS	AHCTF1	252498	2.410902	0.0124
Q3SYF1	Sorting nexin 12	SNX12	18885	0.43094	0.0125

A0A024R9E2	Poly(A) binding protein, cytoplasmic 1, isoform CRA_c	PABPC1	47335	0.802828	0.0125
A0A024R1N1	Myosin, heavy polypeptide 9, non-muscle, isoform CRA_a	MYH9	226532	0.828418	0.0125
Q9Y536	Peptidyl-prolyl cis-trans isomerase A-like 4A	PPIAL4A	18182	0.471714	0.0126
A0A0C4DGB5	Calpastatin	CAST	80999	0.623924	0.0126
D3DVF0	F11 receptor, isoform CRA_a	F11R	32228	3.759321	0.0126
Q8TDN6	Ribosome biogenesis protein BRX1 homolog	BRIX1	41401	1.722353	0.0126
D3DUW5	Dynamin 1-like, isoform CRA_c	DNM1L	87812	0.624942	0.0126
Q9BXP5	Serrate RNA effector molecule homolog	SRRT	100666	0.399194	0.0126
E7EX17	Eukaryotic translation initiation factor 4B	EIF4B	69698	0.556639	0.0126
Q12904	Aminoacyl tRNA synthase complex-interacting multifunctional protein 1	AIMP1	34353	0.737225	0.0127
P02545	Prelamin-A/C	LMNA	74139	1.261804	0.0128
Q9UJY1	Heat shock protein beta-8	HSPB8	21604	3.566249	0.0128
A0A087WWT3	Serum albumin	ALB	45148	0.407978	0.0129
P26232	Catenin alpha-2	CTNNA2	105313	0.604612	0.0129
R4GMT7	Transforming acidic coiled-coil-containing protein 1	TACC1	62407	0.377493	0.0129
D6RCM3	Matrin-3	MATR3	10075	1.725541	0.0129
P49773	Histidine triad nucleotide-binding protein 1	HINT1	13802	0.730008	0.0130
Q6FI81	Anamorsin	CIAPIN1	33582	0.654091	0.0130
Q6NVV1	Putative 60S ribosomal protein L13a protein RPL13AP3	RPL13AP3	12135	0.621701	0.0130
Q9NR45	Sialic acid synthase	NANS	40308	0.307274	0.0130
V9HW39	Epididymis secretory protein Li 100	HEL-S-100	40322	0.307274	0.0130
A0A024R0Q7	Serine/threonine-protein phosphatase	PPP5C	56879	0.351036	0.0130
Q9UMX0	Ubiquilin-1	UBQLN1	62519	0.782906	0.0130
O95036	Similar to 60S ribosomal protein L7;	WUGSC:H_RG054D04.1	29037	0.60736	0.0130

	similar to P18124 (PID:d133021)				
A0A024RB01	Integrin, alpha 5 (Fibronectin receptor, alpha polypeptide), isoform CRA_b	ITGA5	119239	2.215207	0.0130
Q6FG99	RPLP1 protein	RPLP1	11568	0.580875	0.0130
B1ALC0	Actin-related protein 2/3 complex subunit 5	ARPC5	14808	0.308504	0.0132
Q4G0J3	La-related protein 7	LARP7	66899	0.756393	0.0132
O95881	Thioredoxin domain-containing protein 12	TXNDC12	19206	1.492642	0.0133
Q76FK4	Nucleolar protein 8	NOL8	131616	2.071728	0.0134
P35268	60S ribosomal protein L22	RPL22	14787	0.681029	0.0135
A0A024R1P2	Ras-related C3 botulinum toxin substrate 2 (Rho family, small GTP binding protein Rac2), isoform CRA_a	RAC2	21429	0.463271	0.0135
P49903	Selenide, water dikinase 1	SEPHS1	42911	0.649787	0.0138
M0QXH0	Thioredoxin, mitochondrial	TXN2	7027	1.486005	0.0139
A0A0A0MQW1	Kinase suppressor of Ras 1	KSR1	94393	0.278103	0.0139
Q96HQ2	CDKN2AIP N-terminal-like protein	CDKN2AIPNL	13196	0.21963	0.0139
J3KMY0	Cell division cycle-associated protein 3	CDCA3	26262	2.020662	0.0140
A0A024R1A3	Testicular secretory protein Li 63	UBE1	117849	0.43076	0.0140
P22314	Ubiquitin-like modifier-activating enzyme 1	UBA1	117849	0.43076	0.0140
Q5JR95	40S ribosomal protein S8	RPS8	21880	0.718054	0.0141
Q63HR1	Putative uncharacterized protein DKFZp686P17171	DKFZp686P17171	42441	0.683992	0.0141
Q14676	Mediator of DNA damage checkpoint protein 1	MDC1	226666	2.428821	0.0141
Q9Y266	Nuclear migration protein nudC	NUDC	38243	0.215187	0.0144
F5H7X1	26S proteasome non-ATPase regulatory subunit 9	PSMD9	9430	0.623577	0.0144
P30405	Peptidyl-prolyl cis-trans isomerase F, mitochondrial	PPIF	22040	1.165081	0.0144

Q99729	Heterogeneous nuclear ribonucleoprotein A/B	HNRNPAB	36225	1.338515	0.0146
M0QZR9	ELAV-like protein 1	ELAVL1	17131	1.220608	0.0147
O00571	ATP-dependent RNA helicase DDX3X	DDX3X	73243	0.404217	0.0147
P23284	Peptidyl-prolyl cis-trans isomerase B	PPIB	23743	1.181342	0.0148
A0A0G2JP90	Nodal modulator 1	NOMO1	122180	3.124366	0.0148
Q8NBJ5	Procollagen galactosyltransferase 1	COLGALT1	71636	1.850792	0.0148
Q9H814	Phosphorylated adapter RNA export protein	PHAX	44403	0.267003	0.0150
Q5TEC6	Histone H3	H3-7	15430	1.806244	0.0150
Q5IRN2	DAZAP1/MEF2D fusion protein	DAZAP1/MEF2D fusion	48908	0.752436	0.0150
F5GZI0	4F2 cell-surface antigen heavy chain	SLC3A2	18950	1.523952	0.0150
B5MC98	Prolactin regulatory element-binding protein	PREB	38940	3.36634	0.0150
A0A024R5M9	Nuclear mitotic apparatus protein 1, isoform CRA_a	NUMA1	236516	1.328671	0.0150
P68431	Histone H3.1	H3C1	15404	1.870872	0.0150
A0A172WBW8	Complement decay-accelerating factor	CD55	41427	0.60323	0.0150
O14732	Inositol monophosphatase 2	IMPA2	31321	0.269121	0.0151
P0DME0	Protein SETSIP	SETSIP	34882	0.656093	0.0151
O60739	Eukaryotic translation initiation factor 1b	EIF1B	12824	0.701235	0.0151
A0A024RAG3	V-ral simian leukemia viral oncogene homolog B (Ras related GTP binding protein), isoform CRA_a	RALB	23409	2.765968	0.0151
Q5JSZ5	Protein PRRC2B	PRRC2B	242964	0.235739	0.0151
Q15056	Eukaryotic translation initiation factor 4H	EIF4H	27385	0.558522	0.0152
D3DUE6	Cytokine-like nuclear factor n-pac, isoform CRA_c	N-PAC	60986	1.682644	0.0152
Q49A26	Putative oxidoreductase GLYR1	GLYR1	60556	1.682644	0.0152
Q9Y5Z4	Heme-binding protein 2	HEBP2	22875	0.642334	0.0152
F8WD15	Insulin-like growth factor 2 mRNA-	IGF2BP3	8893	0.683211	0.0152

	binding protein 3				
Q13242	Serine/arginine-rich splicing factor 9	SRSF9	25542	1.5867	0.0152
Q04760	Lactoylglutathione lyase	GLO1	20778	0.533011	0.0152
F8VQR7	Cysteine and glycine-rich protein 2	CSRP2	20678	0.554953	0.0155
K7EPF9	Apolipoprotein C-I	APOC1	14274	3.345328	0.0155
P23769	Endothelial transcription factor GATA-2	GATA2	50500	3.345328	0.0155
Q7L0Y3	Mitochondrial ribonuclease P protein 1	TRMT10C	47347	1.376172	0.0159
A6NIZ1	Ras-related protein Rap-1b-like protein	RAP1BL	20925	0.665625	0.0159
Q96HY6	DDRGK domain-containing protein 1	DDRGK1	35611	1.379457	0.0159
Q96ST3	Paired amphipathic helix protein Sin3a	SIN3A	145175	0.767071	0.0159
B7Z1U7	RNA binding protein fox-1 homolog	RBFOX1	45950	0.46708	0.0159
O43251	RNA binding protein fox-1 homolog 2	RBFOX2	41374	0.46708	0.0159
Q8TAE8	Growth arrest and DNA damage-inducible proteins-interacting protein 1	GADD45GIP1	25384	1.344362	0.0160
A0A024R563	Protein phosphatase 1, regulatory (Inhibitor) subunit 14B, isoform CRA_a	PPP1R14B	20965	0.536699	0.0160
O94804	Serine/threonine-protein kinase 10	STK10	112135	0.386273	0.0160
Q14566	DNA replication licensing factor MCM6	MCM6	92889	0.175665	0.0162
O94992	Protein HEXIM1	HEXIM1	40623	3.276153	0.0163
Q96CM8	Acyl-CoA synthetase family member 2, mitochondrial	ACSF2	68125	1.53341	0.0164
J3KSI6	Desmoglein-2	DSG2	25939	3.35296	0.0164
A0A024R7F4	Deoxyribonuclease II, lysosomal, isoform CRA_a	DNASE2	39581	1.490519	0.0164
Q15785	Mitochondrial import receptor subunit TOM34	TOMM34	34559	0.607016	0.0164
Q8TCD5	5'(3')-deoxyribonucleotidase, cytosolic type	NT5C	23383	1.885296	0.0164
V9HWF3	Epididymis luminal protein 74	HEL74	23383	1.885296	0.0164
Q13802	ArgBPIB protein	argBPIB	31183	0.260736	0.0164
Q9NYB9	Abl interactor 2	ABI2	55663	0.260736	0.0164

A0A024R3W7	Eukaryotic translation elongation factor 1 beta 2, isoform CRA_a	EEF1B2	24764	0.773096	0.0165
O60361	Putative nucleoside diphosphate kinase	NME2P1	15529	0.723103	0.0167
Q15691	Microtubule-associated protein RP/EB family member 1	MAPRE1	29999	0.861871	0.0167
O76024	Wolframin	WFS1	100292	1.520213	0.0167
A0A024RB16	Family with sequence similarity 62 (C2 domain containing), member A, isoform CRA_a	FAM62A	124003	4.048206	0.0167
Q9BSJ8	Extended synaptotagmin-1	ESYT1	122856	4.048206	0.0167
A0A024R4D1	COP9 constitutive photomorphogenic homolog subunit 8 (Arabidopsis), isoform CRA_a	COPS8	23226	0.43145	0.0167
P41567	Eukaryotic translation initiation factor 1	EIF1	12732	0.706051	0.0167
Q6IAV3	Eukaryotic translation initiation factor 1, isoform CRA_a	SUI1	12732	0.706051	0.0167
P09496	Clathrin light chain A	CLTA	27077	0.592585	0.0167
A8K517	Ribosomal protein S23, isoform CRA_a	RPS23	15808	0.465196	0.0168
A0A0S2Z4Q6	Hydroxysteroid dehydrogenase 4 isoform 4	HSD17B4	9109	1.866783	0.0169
E5RJR5	S-phase kinase-associated protein 1	SKP1	18720	0.715163	0.0170
Q02809	Procollagen-lysine,2-oxoglutarate 5-dioxygenase 1	PLOD1	83550	2.266845	0.0170
E9PLL6	60S ribosomal protein L27a	RPL27A	12201	0.759101	0.0170
Q9BQQ5	Ribosomal protein L27a	L27a	12015	0.759101	0.0170
O95817	BAG family molecular chaperone regulator 3	BAG3	61595	0.490913	0.0171
Q99990	Transcription cofactor vestigial-like protein 1	VGLL1	28707	0.184427	0.0171
O75533	Splicing factor 3B subunit 1	SF3B1	145830	1.375192	0.0171
Q68D11	Putative uncharacterized protein DKFZp686K23100	DKFZp686K23100	94466	1.328902	0.0174
Q9BYD6	39S ribosomal protein L1, mitochondrial	MRPL1	36909	1.754523	0.0174

A0A024R6Q1	Eukaryotic translation initiation factor 5, isoform CRA_b	EIF5	49223	0.460512	0.0174
Q15428	Splicing factor 3A subunit 2	SF3A2	49256	0.787785	0.0176
Q01813	ATP-dependent 6-phosphofructokinase, platelet type	PFKP	85596	0.514954	0.0178
P21291	Cysteine and glycine-rich protein 1	CSRP1	20567	0.579503	0.0179
Q9BY32	Inosine triphosphate pyrophosphatase	ITPA	21446	0.360938	0.0180
Q549N0	Cofilin 2 (Muscle), isoform CRA_a	CFL2	18737	0.798475	0.0180
P30044	Peroxiredoxin-5, mitochondrial	PRDX5	22086	1.252781	0.0181
B7Z7Q6	cDNA FLJ57232, highly similar to Lysosomal Pro-X carboxypeptidase (EC3.4.16.2)	PRCP	43934	3.370035	0.0182
Q9BRX5	DNA replication complex GINS protein PSF3	GINS3	24535	0.473132	0.0186
P42566	Epidermal growth factor receptor substrate 15	EPS15	98656	0.427323	0.0186
A0A024R6T8	NEFA-interacting nuclear protein NIP30, isoform CRA_a	NIP30	28912	0.336641	0.0186
P45974	Ubiquitin carboxyl-terminal hydrolase 5	USP5	95786	0.628125	0.0186
A0A1B0GUC3	Alpha-ketoglutarate-dependent dioxygenase FTO	FTO	51803	0.464677	0.0186
A0A024QZJ7	Coiled-coil domain containing 6, isoform CRA_a	CCDC6	53295	0.488934	0.0187
B5MCP9	40S ribosomal protein S7	RPS7	21312	0.701247	0.0187
P52815	39S ribosomal protein L12, mitochondrial	MRPL12	21348	1.278252	0.0187
B0I1S9	MYO1B variant protein	MYO1B	131985	0.291697	0.0188
O43765	Small glutamine-rich tetratricopeptide repeat-containing protein alpha	SGTA	34063	0.443044	0.0188
A0A024RDM4	Mitochondrial ribosomal protein 63, isoform CRA_a	MRP63	12266	3.300348	0.0190
P35270	Sepiapterin reductase	SPR	28048	3.300348	0.0190
Q9BQC6	Ribosomal protein 63, mitochondrial	MRPL57	12266	3.300348	0.0190

H3BM42	Golgi apparatus protein 1	GLG1	79317	1.74793	0.0192
Q92575	UBX domain-containing protein 4	UBXN4	56778	4.199674	0.0193
Q147Y3	PABPC4 protein	PABPC4	27900	0.620896	0.0194
Q9NQP4	Prefoldin subunit 4	PFDN4	15314	0.46766	0.0194
P62937	Peptidyl-prolyl cis-trans isomerase A	PPIA	18012	0.717114	0.0195
A0A024R7I5	TRM1 tRNA methyltransferase 1 homolog (S. cerevisiae), isoform CRA_c	TRMT1	72234	0.466537	0.0195
A0A024R693	Galectin	hCG_22119	26152	0.746859	0.0198
P17931	Galectin-3	LGALS3	26152	0.746859	0.0198
Q53XJ5	Peptidylprolyl isomerase	FKBP2	15649	1.572551	0.0198
D3DX26	RAN binding protein 1, isoform CRA_b	RANBP1	17557	0.686735	0.0198
D3DQH8	Secreted protein, acidic, cysteine-rich (Osteonectin), isoform CRA_a	SPARC	36051	1.34275	0.0199
P51665	26S proteasome non-ATPase regulatory subunit 7	PSMD7	37025	0.462358	0.0200
Q9UBS4	DnaJ homolog subfamily B member 11	DNAJB11	40514	1.27896	0.0201
P30101	Protein disulfide-isomerase A3	PDIA3	56782	1.324231	0.0201
A0A090N7V5	Chromosome 7 open reading frame 24	C7orf24	21008	0.314215	0.0201
O75223	Gamma-glutamylcyclotransferase	GGCT	21008	0.314215	0.0201
Q9BZQ6	ER degradation-enhancing alpha-mannosidase-like protein 3	EDEM3	104664	1.622154	0.0204
B2R6S5	UMP-CMP kinase	CMPK1	25855	0.781025	0.0212
P07305	Histone H1.0	H1-0	20863	1.642616	0.0214
A0A024R4S1	Epsin 1, isoform CRA_a	EPN1	60293	1.482026	0.0214
Q99614	Tetratricopeptide repeat protein 1	TTC1	33526	0.619546	0.0214
O15212	Prefoldin subunit 6	PFDN6	14583	0.705602	0.0215
O60437	Periplakin	PPL	204747	2.310439	0.0216
A0A140VJQ8	Paxillin, isoform CRA_e	PXN	64505	0.759203	0.0217
P20290	Transcription factor BTF3	BTF3	22168	0.473677	0.0221
A0A024R152	HCG28765, isoform CRA_b	hCG_28765	147227	0.626817	0.0223
Q9Y2S0	DNA-directed RNA polymerases I and	POLR1D	15237	0.623388	0.0224

	III subunit RPAC2				
A0A024RD03	Mitochondrial ribosomal protein S10, isoform CRA_a	MRPS10	22999	1.84062	0.0227
P42696	RNA-binding protein 34	RBM34	48565	1.474797	0.0229
Q9H072	Putative uncharacterized protein DKFZp586J151	DKFZp586J151	58095	1.69638	0.0232
O75330	Hyaluronan mediated motility receptor	HMMR	84100	0.657679	0.0242
A0A0A0MT33	Protein SCAF8	SCAF8	148859	0.46197	0.0253
A0A024R1M8	Apolipoprotein L, 2, isoform CRA_a	APOL2	37078	0.682532	0.0255
E5RHG6	Tubulin-specific chaperone A	TBCA	15062	0.764251	0.0263
Q9UGM6	Tryptophan--tRNA ligase, mitochondrial	WARS2	40147	2.610436	0.0263
Q06830	Peroxiredoxin-1	PRDX1	22110	0.816592	0.0271
F8VY02	Endoplasmic reticulum resident protein 29	ERP29	18116	1.249485	0.0277
A0M8W4	Ubiquitin-conjugating enzyme E2 variant 2	UBE2V2	16363	0.56364	0.0279
V9HW35	Epididymis secretory protein Li 55	HEL-S-55	17031	1.260077	0.0279
A4D2P0	Ras-related C3 botulinum toxin substrate 1 (Rho family, small GTP binding protein Rac1)	RAC1	23467	0.501585	0.0279
A0A024R663	Kinectin 1 (Kinesin receptor), isoform CRA_a	KTN1	156275	1.165821	0.0283
D3DS95	HCG21173, isoform CRA_a	hCG_21173	10123	0.756263	0.0287
K7EQ03	RNA-binding protein 42	RBM42	44638	0.756263	0.0287
D3DV75	Adenosine deaminase, RNA-specific, isoform CRA_b	ADAR	98746	1.177405	0.0287
C9J660	Sulfatase-modifying factor 2	SUMF2	39746	1.32345	0.0305
Q9NUP9	Protein lin-7 homolog C	LIN7C	21834	0.669826	0.0305
P09012	U1 small nuclear ribonucleoprotein A	SNRPA	31280	0.693874	0.0307
B3GUD5	Protein tyrosine phosphatase-2	PTPN11	24119	0.715486	0.0310
A0A024RAF2	Diazepam binding inhibitor	DBI	11793	0.803718	0.0312
Q86SQ0	Pleckstrin homology-like domain family	PHLDB2	142158	0.460766	0.0316

	B member 2				
G8JLB6	Heterogeneous nuclear ribonucleoprotein H	HNRNPH1	51230	0.752117	0.0326
Q5T8U2	60S ribosomal protein L7a	RPL7A	16540	0.700877	0.0329
Q15293	Reticulocalbin-1	RCN1	38890	1.258205	0.0337
V9HW95	Epididymis secretory protein Li 84	HEL-S-84	38890	1.258205	0.0337
A0A024RDP4	Paraspeckle component 1, isoform CRA_b	PSPC1	45571	0.783044	0.0356
H3BT13	Small nuclear ribonucleoprotein Sm D3	SNRPD3	6906	0.819215	0.0381
Q15942	Zyxin	ZYX	61277	0.678308	0.0397
B3KM80	Nucleolin, isoform CRA_c	NCL	58555	1.124455	0.0398
J3KT29	60S ribosomal protein L23	RPL23	13193	0.847902	0.0398
K7ELW0	Protein deglycase DJ-1	PARK7	17910	0.813511	0.0416
Q96EP5	DAZ-associated protein 1	DAZAP1	43383	0.816083	0.0441
Q04837	Single-stranded DNA-binding protein, mitochondrial	SSBP1	17260	1.11243	0.0452
A0A024R7M0	Transmembrane emp24 protein transport domain containing 9, isoform CRA_a	TMED9	27277	1.290593	0.0452
