# Supplementary material for: A Proteomics-Based Identification of the Biological Networks Mediating the Impact of Epigallocatechin-3-Gallate on Trophoblast Cell Migration and Invasion, with Potential Implications for Maternal and Fetal Health
Source: Proteomes. 2023 Oct 12;11(4):31. doi: 10.3390/proteomes11040031 (PMC10594419; doi:10.3390/proteomes11040031)
Supplement: Supplementary file 1 [file proteomes-11-00031-s001.zip › Supplementary Table S1.pdf]

Table S1: List of primers used in Quantitative Real-Time PCR

Gene ID	Protein Accession Number	Protein Name	Forward Primer	Reverse Primer
ACAA2	K7EME0	acetyl-CoA acyltransferase 2	GGGCACTGAAGAAAGCAGGA	CGTGAACCAGGTGTGCAGTA
RAB7A	A0A158RFU6	RAB7A, member RAS oncogene family	TGGGAGATTCTGGAGTCGGG	CTGTAGAAGGCCACACCGAG
ACADVL	B3KPA6	acyl-CoA dehydrogenase very long chain	CCGGAGAGATTCGGAGATGC	TCAGAGGGGTGGGAATCTGA
ABI2	Q9NYB9	abl interactor 2	CAGTGGGAGTAGTGGAGGGA	CAGGAACAAGAGGAGCAGGG
ATP5F1D	P30049	ATP synthase F1 subunit delta	CGGCACCACCTCCAAATACT	CCTGGGCCTTCTCCAAGTTT
CRAT	P43155	carnitine O-acetyltransferase	AGGCTCTAGCAAGGACCCA	TCAAGGAGAAGGGCTTCAGG
ABHD10	Q9NUJ1	abhydrolase domain containing 10	AGCTGATGGGCCACAGATTC	GCATGCTCCACACACCTTTC
PRDX5	P30044	peroxiredoxin 5	GGACTCTCAGTTCACCCACC	AGTGATCTGGCGAAGGACAC
CES1	P23141	carboxylesterase 1	GGACTTACAGGGAGACCCCA	TGCATTGGAATCAACCAGCCA
F11R	Q6FIB4	F11 receptor	ACAGTGCACTCTTCTGAACCT	TGGCAGAGGAGGGGATGTTA
