# Supplementary figures and images for: A Proteomics-Based Identification of the Biological Networks Mediating the Impact of Epigallocatechin-3-Gallate on Trophoblast Cell Migration and Invasion, with Potential Implications for Maternal and Fetal Health
Source: Proteomes. 2023 Oct 12;11(4):31. doi: 10.3390/proteomes11040031 (PMC10594419; doi:10.3390/proteomes11040031)

# Cell Cycle

## DNA Replication

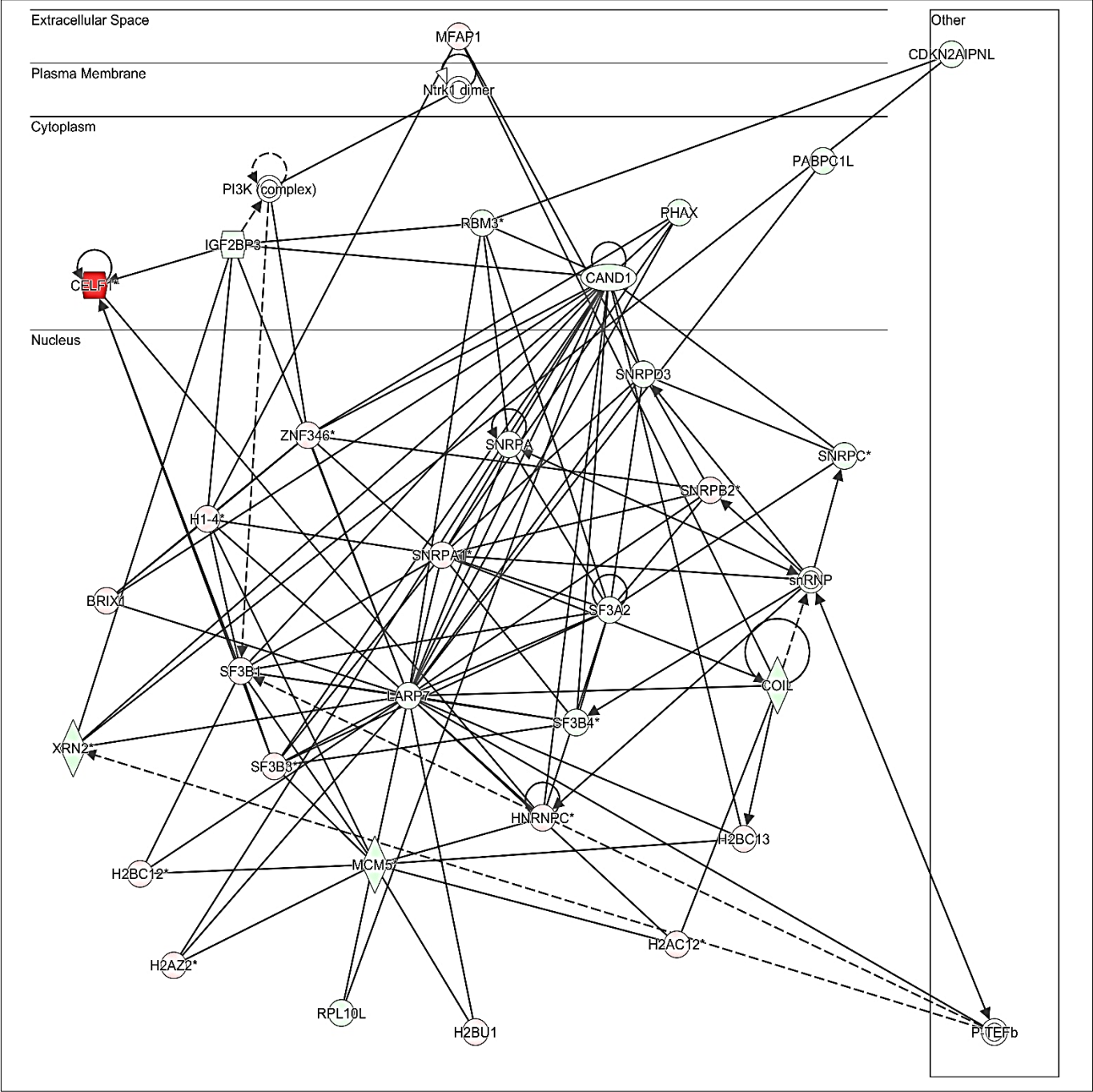

# Energy Production

## Protein Synthesis

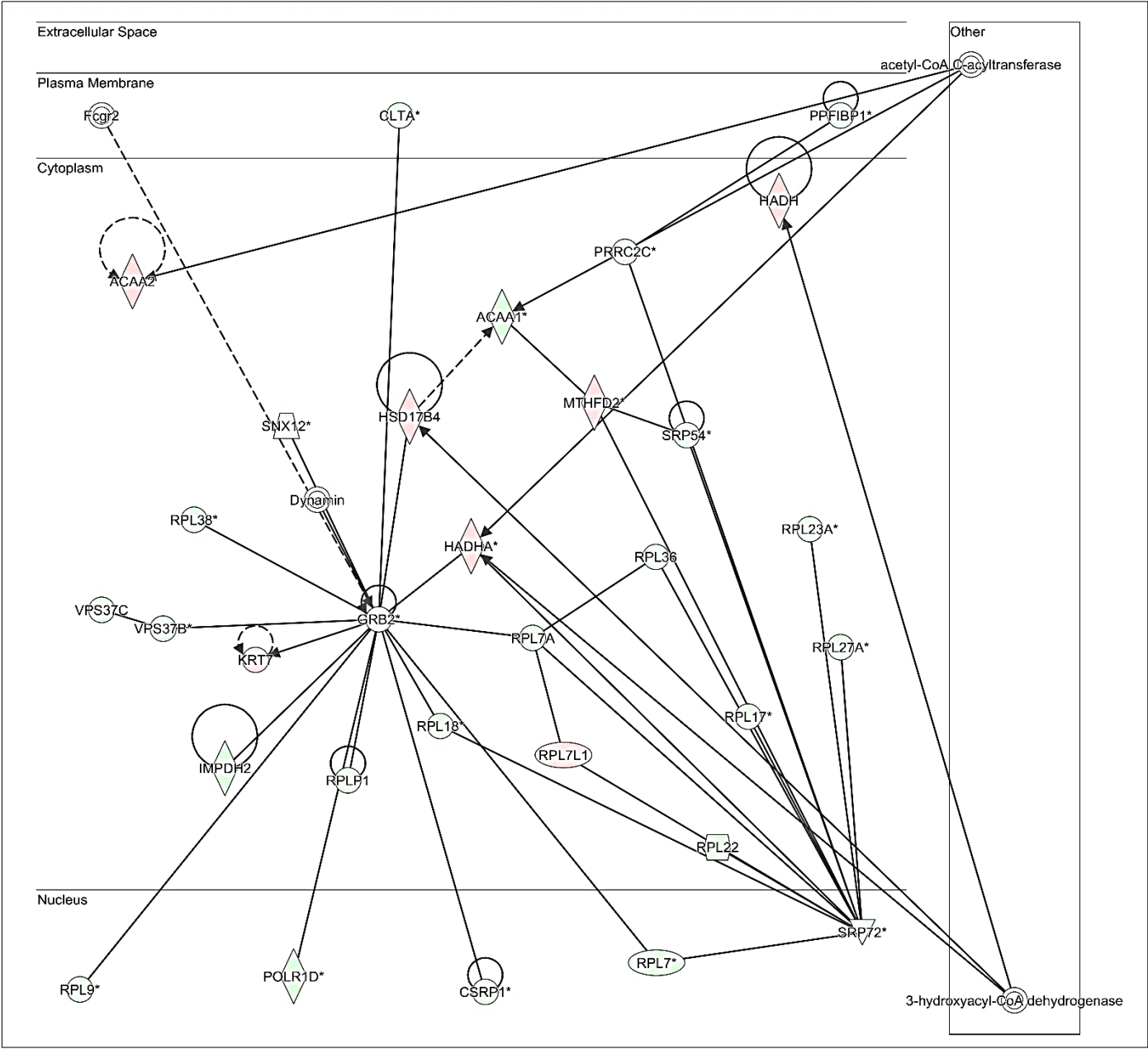



# Connective Tissue

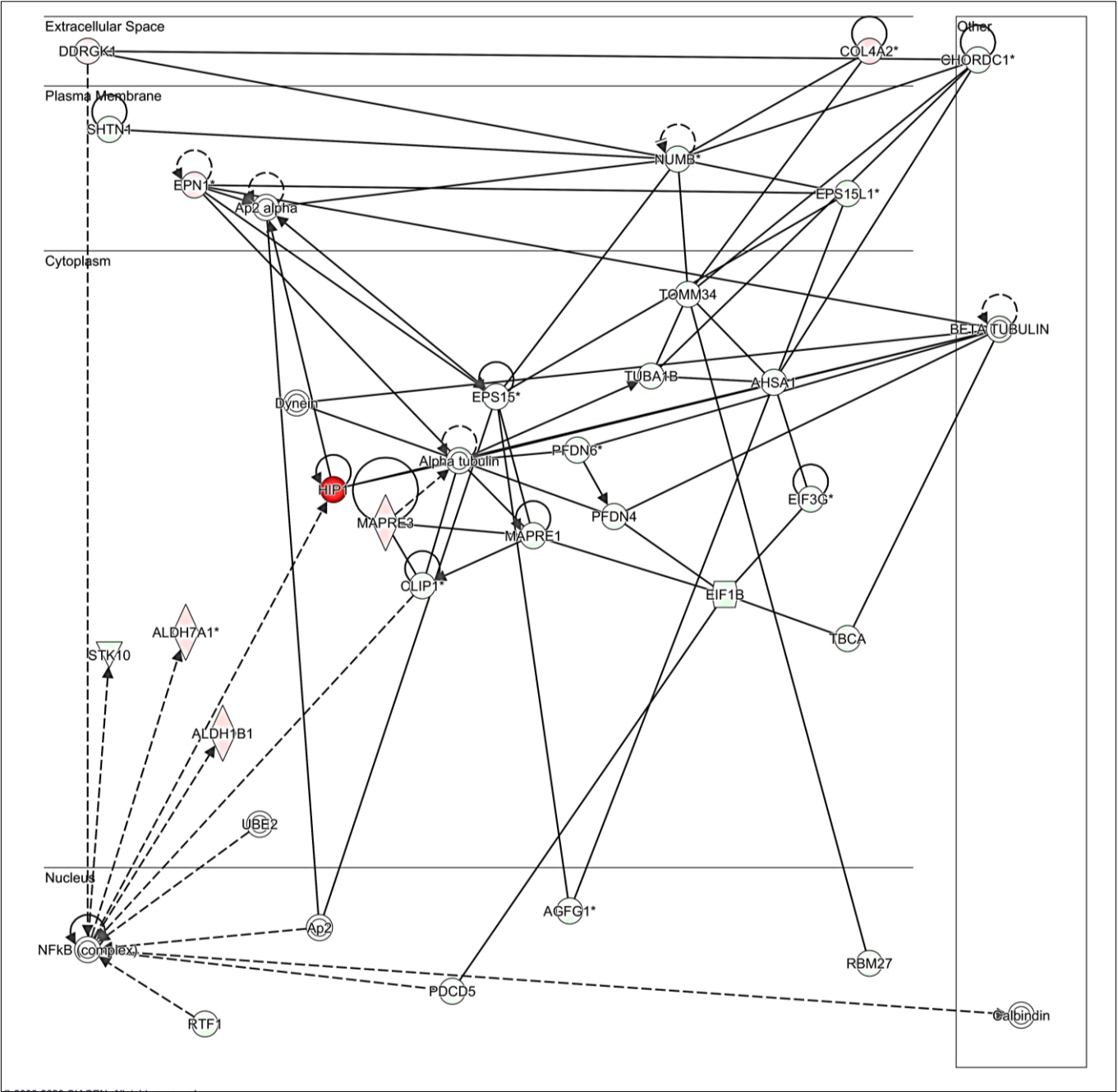

Supplement: Supplementary file 1 [file proteomes-11-00031-s001.zip › Supplementary Figure S1.pdf]
